# Surfactant-Assisted Synthesis of Metallic-Ag/Nickel Oxide on Graphitic Carbon Nitride Composite: An Electrochemical Investigation of Synthetic Vanillin

**DOI:** 10.1021/acsami.4c19099

**Published:** 2025-02-06

**Authors:** Muthukumar Govindaraj, Balasubramanian Sriram, Sea-Fue Wang, Magesh Kumar Muthukumaran, Sakthivel Kogularasu, Guo-Ping Chang-Chien, Arockia Selvi J.

**Affiliations:** † Department of Chemistry, SRM Institute of Science and Technology, Kattankulathur-603203, Tamil Nadu, India; ‡ Department of Materials and Mineral Resources Engineering, National Taipei University of Technology, Taipei 106, Taiwan; § Super Micro Mass Research and Technology Center, Cheng Shiu University, Kaohsiung 833301, Taiwan; ∥ Center for Environmental Toxin and Emerging-Contaminant Research, Cheng Shiu University, Kaohsiung 833301, Taiwan; ⊥ Institute of Environmental Toxin and Emerging-Contaminant, Cheng Shiu University, Kaohsiung 833301, Taiwan

**Keywords:** vanillin, nickel oxide, food samples, electrocatalyst, electrochemical sensor

## Abstract

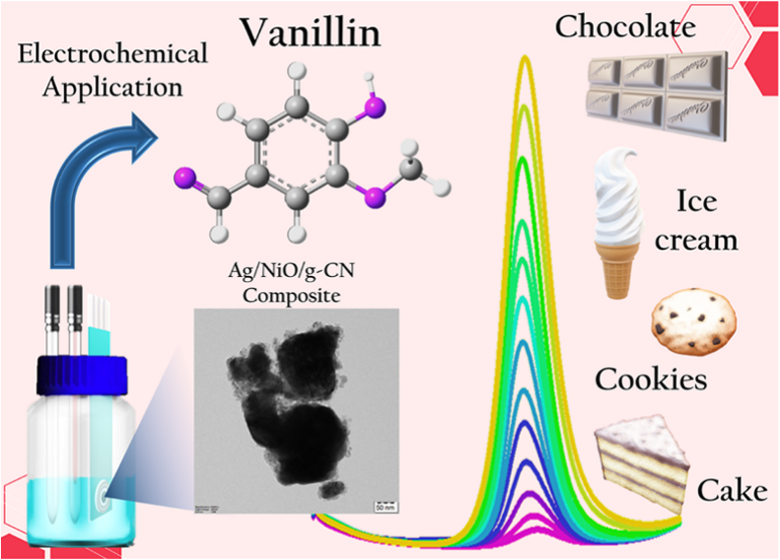

In this study, we developed a sensor based on surfactant-assisted synthesis of metallic silver-enriched nickel oxide confined on graphitic carbon nitride (Ag/NiO/g-CN)-modified electrode to construct a sensitive and selective voltammetric sensor for detecting vanillin in confectionaries samples. The X-ray diffraction (XRD) and Fourier transform infrared (FT-IR) spectroscopy analyses confirmed the crystal structure and respective functional groups of the synthesized Ag/NiO/g-CN composite. The valence states of silver, nickel, oxygen, carbon, and nitrogen were analyzed using X-ray photoelectron spectroscopy (XPS), while energy-dispersive X-ray analysis (EDX) and morphological investigations revealed the elemental distribution and nano-structured particles, respectively. The electrocatalyst-modified electrode properties and electrochemical sensing performances were evaluated using different voltammetric and spectroscopic techniques. The Ag/NiO/g-CN composite, exhibiting a large active surface area, excellent conductivity, and synergistic interaction, proved to be a suitable electrode material for electrochemical sensor applications. The sensor demonstrated a detection limit of 0.9 nM and a broad linear range of 0.004–366.8 μM. Electrochemical investigations further highlighted the sensor’s excellent reproducibility, repeatability, fast response, and functional stability. The constructed sensor also exhibited outstanding selectivity against potential interferents and demonstrated its practical applicability by successfully detecting vanillin in spiked food samples.

## Introduction

The primary aromatic component of natural vanilla is vanillin (4-hydroxy-3-methoxybenzaldehyde). Vanillin is employed extensively in food and nonfood industries because of its distinct flavor and aroma.^1,2^ The secondary metabolism of fruits, plants, and vegetables is produced from the phenolic compound vanillin, and it possesses antioxidant properties and it is crucial in preventing degenerative illnesses such as cancer and atherosclerosis.^3−5^ Owing to its significant physiological characteristics, vanillin is frequently used in the culinary and pharmaceutical industries as a component of various products.^6−8^ For instance, it is a spice in pharmaceutical preparations, a fragrance ingredient in perfumes and cosmetics, and a culinary additive to enhance the flavor of custard, cookies, chocolate, ice cream, and beverages.^9,10^ Vanillin has been shown to have beneficial health impacts, including lowering the death rate from heart disease and the antiadhesion effect of sickle cell anemia by preventing human low-density lipoprotein oxidation.^2,5,11^ Vanillin’s chemical characteristics make it worthwhile for the pharmaceutical industry’s creation of antiophidic serums. Nevertheless, natural vanillin is extremely expensive and produced from vanilla pods barely meets 0.2% of market demand.^9,12−14^ Eugenol, lignin, and 2-methoxyphenol are inexpensive essential ingredients chemically synthesized to produce vanillin. Synthetic vanillin is cheap and highly productive, but when taken in excess, it can lead to headaches, nausea, and vomiting, as well as problems with the liver and kidneys.^15−19^ Therefore, regulating the amount of vanillin in food is crucial to ensure food safety.

The requirement for appropriate analytical techniques for the practical identification and quantification of this phenolic component in complex matrices has arisen from the extensive use of vanillin in formulating numerous products in the food and pharmaceutical industries. Even though food and pharmaceutical businesses use vanillin to produce many different goods, it is evident that developing such analytical methods for vanillin quantification is fundamentally vital for public health policy.^20−22^ According to the published research, gas chromatography, liquid chromatography, high-performance liquid chromatography, capillary electrophoresis, thin-layer chromatography, UV–Vis spectrophotometry, chemiluminometry, spectroscopy, micellar electrokinetic chromatography, and electrochemical sensing techniques determine vanillin.^5,12,14,16,22−32^ Most of these techniques produce beneficial information for identification, quantification, and selectivity. At the same time, their sample pretreatment processes are time-consuming and intricate, requiring very advanced equipment. The electrochemical approach^33−38^ offers potential benefits in determining various analytes because of its low cost,^39^ quick reaction,^40,41^ ease of use,^42^ high sensitivity,^43^ and robust detection ability.^44−49^

Nevertheless, issues with electrode surface fouling and high oxidation potential, where oxygen evolution current interferes, restrict the electrochemical study of vanillin. Various chemically modified electrodes have been reported for the detection of vanillin such as graphene (GR), Au–Pd nanoparticles/GR composite, boron-doped diamond, graphene oxide (GR), manganese dioxide nanoflowers–graphene oxide (GO) composite, silver nanoplates/GR composite, graphene–polyvinylpyrrolidone composite, Ag–Pd bimetallic nanoparticles-decorated graphene oxide, electrospun molybdenum disulfide (MoS_2_) nanoparticles–carbon nanofibers composite, cathodically pretreated boron-doped diamond, cadmium oxide nanoparticle-decorated single wall carbon nanotubes, and electrolytic manganese dioxide–graphene composite.^5,12,16,22,29,30,50−55^ The altered material significantly impacts the sensitivity and selectivity of the electrochemical vanillin determination. Therefore, it is essential to investigate new electrode modifications to detect vanillin rapidly and reliably.

Various nanomaterials are reported so far, such as graphene, graphene oxide, carbon nanotubes, conducting polymers, MXenes, and metal oxides (CuO, Cu_2_O, NiO, RuO, Co_3_O_4_, MnO_2_, and V_2_O_5_), for use in several applications such as supercapacitors, batteries, photocatalysts for hydrogen production and degradation, and electrocatalyst for water splitting and electrochemical sensor applications.^56−61^ Most frequently, transition-metal oxides shows higher capacitive behavior than carbon-based materials with limited capacitive properties. Among these materials, nickel oxide is an environmentally benign material with a highly reversible redox nature and has a high capacitive behavior. The electrical conductivity of the materials plays a crucial role in the electrochemical reactions, enhancing the performance of the sensor applications. Although the performance of nickel oxide is limited by its low electrical conductivity, metallic silver has been added to improve its conductivity.^62−64^ This has improved electrolyte ion diffusion, the utilization of electroactive sites in nickel oxide-modified electrodes, and electrical conductivity. Further, the electrochemical active surface area was enhanced using 2D graphitic carbon nitride (g-CN), and it is amine functionalities and lone pair of electrons in the nitrogen atoms were abundant.

Graphitic carbon nitride (g-CN) is another two-dimensional (2D) metal-free polymeric semiconductor type. It comprises nitrogen and carbon atoms joined by planner amino groups in each layer, triazine or tri-s-triazine units, and a weak van der Waals interaction between layers. Owing to its exceptional qualities, including excellent electrical and thermal conductivity, higher active surface area, electronic structure, high chemical stability, and biocompatibility, 2D g-CN has garnered much attention in catalyst research.^65−68^ In the fields of photocatalysis, hydrogen evolution reaction (HER), energy storage conversion, and catalysis, g-CN has drawn much interest. Additionally, too many uniform nitrogen coordinators in the g-CN structure can produce more metal coordination sites, which can serve as catalytic active sites during the electrochemical process and increase the activities for electrocatalytic performance.^69−72^ It can be utilized for sensing applications to find metal ions and biomolecules. Metal oxide and g-CN together have the potential to create heterogeneous nanocomposites with enormous active sites and excellent synergistic effects.

**Scheme 1:**
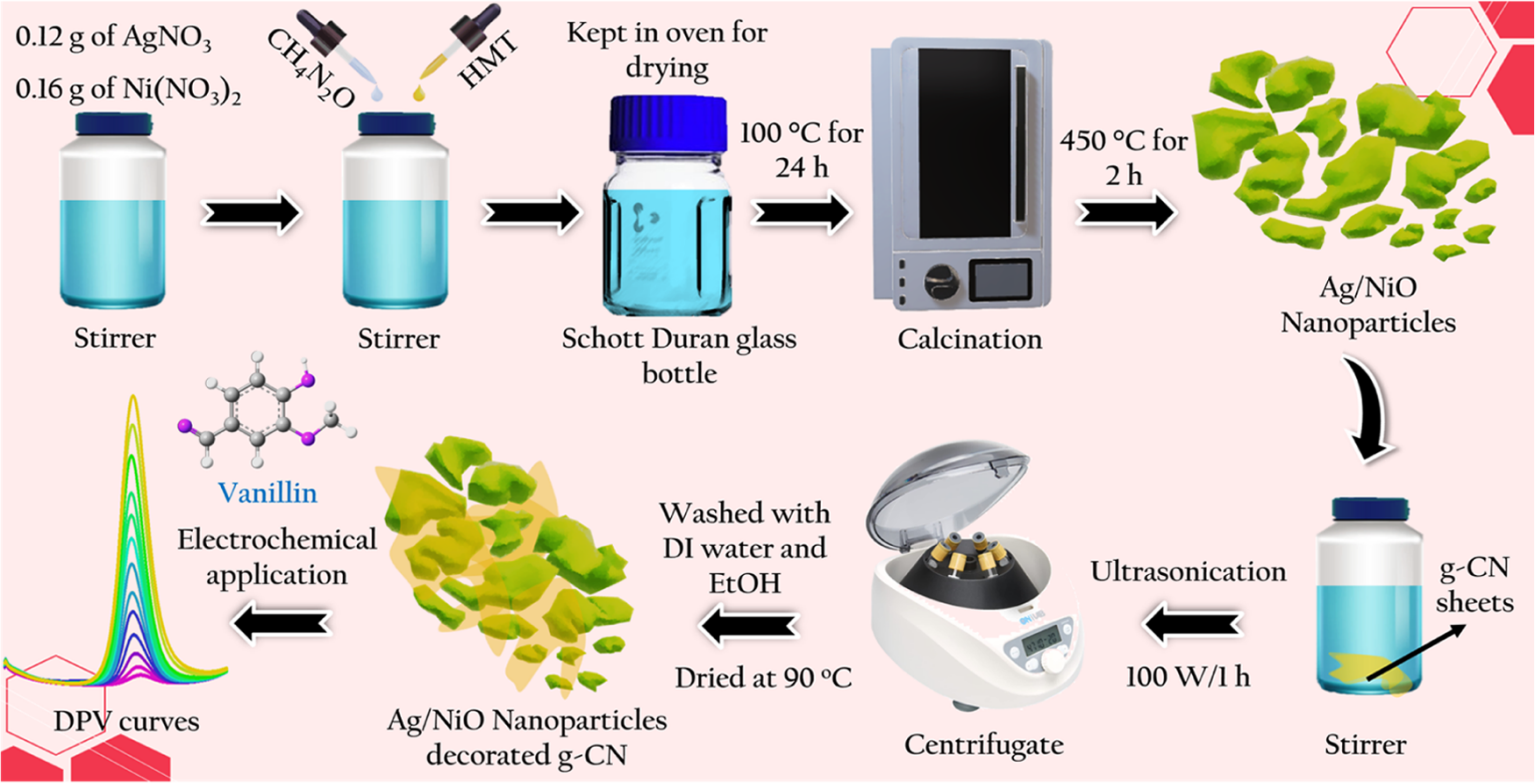
Synthesis of Ag/NiO Nanoparticles and Ag/NiO/g-CN Composite toward the Electrochemical Determination of Vanillin

This study describes the electrochemical performance of the electrode and the single-route surfactant-assisted synthesis of metallic silver nickel oxide on graphitic carbon nitride nanoarray design. Metallic silver peaks combined with NiO/g-CN are seen by X-ray diffraction (XRD). X-ray photoelectron spectroscopy (XPS) analyzed the metallic silver binding energy, revealing silver’s presence in the synthesized NiO nanoparticles. The three-electrode measurements demonstrated Ag/NiO/g-CN composite as an appropriate electrode material for electrochemical sensor applications (Scheme 1).

## Experimental Section

The section on chemicals and material characterization is described in depth in the Supporting Information.

### Synthesis of g-CN

The graphitic carbon nitride (g-CN) was synthesized by one-step thermal polymerization of melamine in a muffle furnace at 550 °C for 3 h (N_2_ atmos.), as described in our previous reports.^44,45^ During the thermal treatment, the discrete oligomers are created by melamine condensation and subsequently evolve into more extended polymeric networks when planar amino groups bind tri-s-triazine molecules together.

### Synthesis of Ag/NiO Nanoparticles

The metallic silver nickel oxide on graphitic carbon nitride nanoarrays was synthesized using a simple surfactant-assisted method. Briefly, 0.12 g of AgNO_3_ and 0.16 g of Ni(NO_3_)_2_·6H_2_O were dissolved in 30 mL of double-distilled water. The two solutions were mixed at a constant stirring for 15 min. Subsequently, 10 mL of HMT was added to the precursor solution, followed by 20 mL of urea solution, which was agitated for 30 min. After the final solution was poured into a Schott Duran glass bottle and sealed with a polypropylene screw top, and kept in the oven at 100 °C for 24 h. The residue was parted by centrifugation and washed thoroughly with ethanol and water several times. After being collected, the samples were desiccated at around 100 °C for a day and then calcined for 2 h at 450 °C at a heating rate of 5 °C per min.

### Synthesis of Ag/NiO/g-CN Nanocomposite

The Ag/NiO/g-CN nanocomposite was synthesized by dispersing Ag/NiO and g-CN in DI water to form a homogeneous solution. The mixture was subjected to ultrasonication at 100 W for 1 h to ensure thorough mixing, prevent agglomeration, and promote uniform distribution of the two materials. Following sonication, the resulting suspension was carefully washed multiple times with DI water to eliminate any residual impurities or unreacted precursors. The purified material was then dried in an oven at 90 °C overnight, allowing for the complete removal of moisture and ensuring the formation of a stable and well-structured nanocomposite. For comparison, Ag/NiO was synthesized under identical conditions, but without the addition of g-CN. This consistent procedure ensures a reliable baseline for evaluating the synergistic effect of g-CN incorporation into the Ag/NiO system. The resulting materials were subsequently characterized and utilized for electrochemical studies.

### Fabrication of Electrodes

According to the working electrode fabrication procedure, the SPCE surface was first cleaned to ensure optimal surface conditions for modification. SPCE was sequentially washed with ethanol and DI water to remove any surface contaminants. This step is critical for achieving uniform material deposition and ensuring reproducible sensor performance.

Following the cleaning process, a solution of the prepared nanomaterials Ag/NiO, g-CN, and the composite Ag/NiO/g-CN was prepared. For this, 2 mg of the respective materials was accurately weighed and dispersed in 1 mL of DI water, resulting in a concentration of 2 mg/mL. To achieve proper dispersion and homogeneity of the nanomaterials, the solution was subjected to ultrasonication for 10 min. Sonication ensures that the nanomaterials are uniformly distributed and free from agglomeration, which is crucial for obtaining a consistent and active electrode surface.

After sonication, 6 μL of each dispersed solution (Ag/NiO, g-CN, and Ag/NiO/g-CN) was carefully drop-cast onto the cleaned SPCE surface. The electrodes were then dried at 60 °C for sufficient time to evaporate the water solvent, ensuring the nanomaterials adhered firmly to the electrode surface. The drying process also aids in forming a stable and uniform film of the nanocomposite on the electrode surface, which is essential for reliable electrochemical performance. The resulting modified electrodes Ag/NiO/SPCE, g-CN/SPCE, and Ag/NiO/g-CN/SPCE were then ready for subsequent electrochemical characterization and vanillin sensing experiments.

## Results and Discussion

### Selection of Material as an Electrocatalyst

Owing to their unique properties, 2D materials have garnered significant interest for their potential broad use. Carbon nitrides’ remarkable multidimensionality and nanostructure-creation characteristics are highly advantageous for electrochemical sensor applications. Its extraordinary strength, electric conductivity, and layer carbon nitride structure all contribute to its exceptional qualities. In a different context, silver nickel oxide’s intriguing characteristics render it a very versatile material with potential applications as a catalyst. The electrochemical performance of Ag/NiO/g-CN heterojunction improved its activity due to its better surface area, active sites, and increased electrical conductivity. The hybrid composite shows enhanced electrocatalyst sensing capability with better parameter analysis.

### Crystalline Properties of Synthesized Nanohybrid

**Figure 1:**
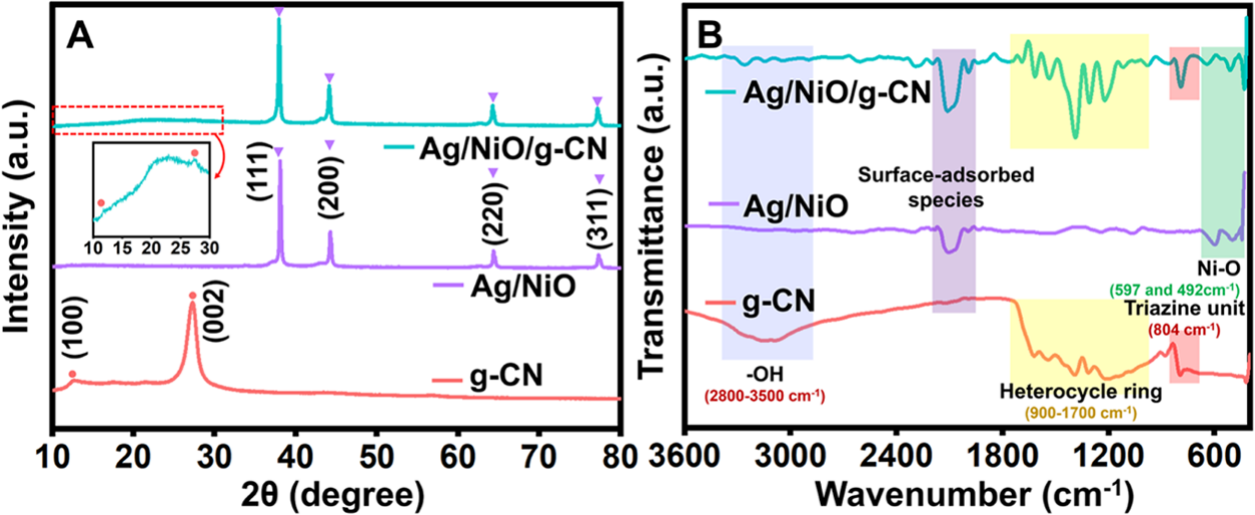
(A) XRD patterns and (B) FT-IR spectra of g-CN, Ag/NiO, and the Ag/NiO/g-CN composite.

The XRD pattern displaying the phase compositions of Ag/NiO, g-CN, and the Ag/NiO/g-CN composite is shown in Figure 1A. The cubic crystal structure of metallic silver with nickel oxide agreed with the diffraction angles. The JCPDS number 01-73-1523 for NiO was found to match with the peaks at 37.22, 43.254, 62.83, and 75.35° 2θ. The metallic silver peaks that corresponded to the JCPDS number 01-87-0719 were found 2θ values at 38.2, 44.4, 64.7, 77.7, and 81.9°. According to this, results confirm the presence of metallic silver with nickel oxide. At the electrode/electrolyte interface, silver increases the NiO nanoarrays’ electrical conductivity, which is better for the electrochemical redox process. The XRD pattern of g-CN, which corresponds with JCPDS number 01-087-1526, has two diffraction peaks identical to g-CN at 13.1 and 27.3° referenced to (100) and (002). The in-planar and interplanar structural stacking of the aromatic rings causes these peaks. On the other hand, all constituent planes inside the single pattern of the Ag/NiO/g-CN hybrid with minor peak shifts suggest the hybrid formed successfully. Scherrer’s equation determined the hybrid’s crystal sizes to be ≈62 nm. The following formulas were used to calculate the mean crystal size (eq 1), dislocation density (eq 2), and the sample’s microstrain (eq 3); results are displayed in Table 1.
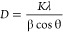
1
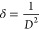
2
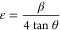
3

**Table 1:** XRD Parameters: Crystallite Size, Dislocation Density, and Microstrain of the Samples

s. no	sample	peak plane	crystallite size *D* (nm)	dislocation density δ (nm^–2^)	microstrain ε (%)
1	Ag/NiO	(111)	62	3.3764	3.28
2	g-CN	(002)	42	7.0175	4.27
3	Ag/NiO/g-CN	(111)	23	4.7146	1.57

In this equation, “*D*” represents the crystallite size, “*K*” represents the crystal lattice constant (0.94), “β” represents the full width at half-maximum (fwhm), “λ” represents the X-ray wavelength (1.5406), “θ” represents an angle of diffraction, “δ” represents the dislocation density, and “ε” represents the microstrain.^45^

### Structural and Chemical Properties of Synthesized Nanohybrid

The FT-IR spectra display the structural and chemical properties of Ag/NiO, g-CN, and the Ag/NiO/g-CN composite, shown in Figure 1B. The FT-IR spectra of g-CN indicate the existence of aromatic CN heterocycles. Stretching vibration patterns of the −NH_2_ bonded to the sp^2^ hybridized carbon or = N–H groups at the aromatic ring’s defect sites show a prominent peak between approximately ≈3100 and ≈3400 cm^**–**1^, suggesting that these groups are uncondensed amines. The stretching vibration modes of −C=N and −C-N heterocycles are shown by the peaks at approximately ≈1240, ≈1325, ≈1420, ≈1560, and ≈1640 cm^**–**1^, respectively. The modest peak at ∼804 cm^**–**1^ indicates the presence of triazine rings in the breathing vibration mode of g-CN. The absorption characteristic at ∼889 cm^**–**1^ was associated with the across-linked heptazine deformation mechanism. Every individual peak agreed with previously reported g-CN synthesized results.^44,45^ Also, the Ni–O stretching vibrations were identified as the source of the spectra peaks occurring at ≈492 and ≈597 cm^**–**1^. At ≈953, ≈1422, and ≈2000 cm^–1^, N–H wagging, −CH_2_ stretching, and C–O modes were identified, which could be caused by surface-adsorbed species.^73^ On the other hand, the Ag/NiO/g-CN hybrid shows all of the individual transmittance vibrations of both Ag/NiO and g-CN in a single spectrum along with a slight peak shift, which indicates the probable interaction among the molecules through the −OH group. In addition, the interaction between Ag/NiO and g-CN caused an internal electric field and a weak van der Waals interaction, forming Ag/NiO on the surface of g-CN.

### Surface Functional and Chemical Properties of Synthesized Nanohybrid

**Figure 2:**
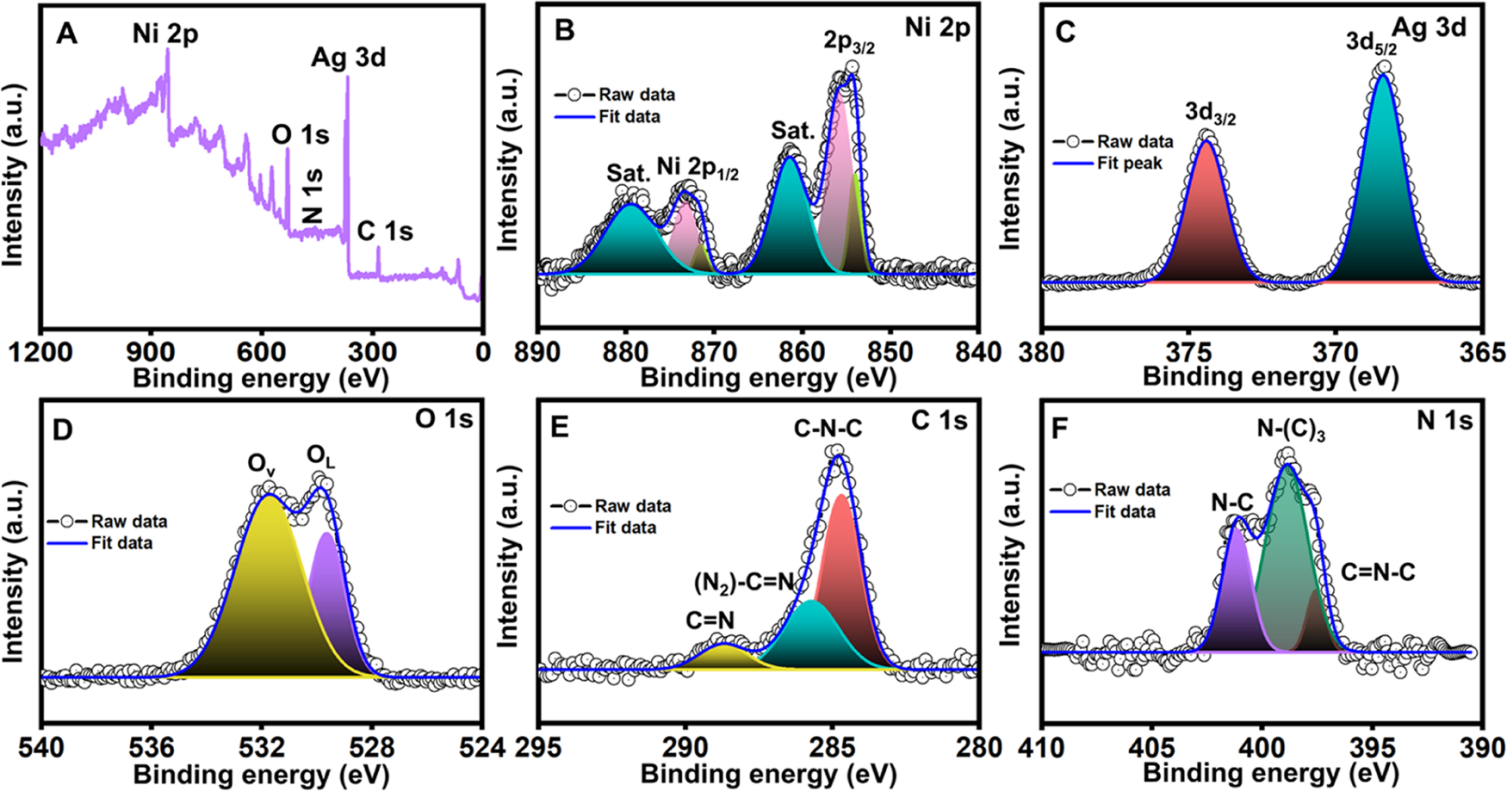
XPS spectrum of Ag/NiO/g-CN composite: (A) overall, (B) Ni 2p, (C) Ag 3d, (D) O 1s, (E) C 1s, and (F) N 1s.

The survey spectrum of the Ag/NiO/g-CN nanohybrid is shown in Figure 2A. Additionally, the high-resolution XPS spectra were used to characterize each element’s valence state quantitatively, emphasizing the integrity of the composite. The intensity increases in tandem with the binding energy. The strong binding energy of the metal increases the difficulty of electron remittance when monochromatic X-rays travel through its surface. The binding energy rises due to the re-emitted electrons’ increasing intensity, and simultaneous inelastic collisions happen. XPS determined silver, nickel, oxygen, carbon, and nitrogen valence. The survey scan verified the material’s existence and its oxidation valence states (Ni 2p, Ag 3d, O 1s, C 1s, and N 1s). Five different peaks in Figure 2B represent the high-resolution spectrum of Ni 2p (Ni^2+^). The Ni 2p_3/2_ state and satellite peak were assigned binding energies of ≈854.1, ≈855.4, and ≈861.4 eV, respectively. Although it has been reported to contain a minor contribution from surface states, the binding energy at 854.1 eV (Ni^2+^) peak attributed to the local screening from lattice oxygen to the Ni 2p core hole and 855.4 eV attributed to the nonlocal screening from the lattice. The Ni 2p_1/2_ state, originating from Ni–O species, was detected at ≈874.1 eV, and its matching satellite peak was detected at ≈879.6 eV.^74^ The deconvoluted high-resolution Ag 3d’s spectra show two peaks at ≈368.3 and ≈374.4 eV, which correspond to Ag 3d_5/2_ and Ag 3d_3/2_, respectively (Figure 2C), and were attributed to the former and latter peaks, indicating the existence of metallic silver in the Ag/NiO nanoarrays.^75^ The oxygen high-resolution spectrum is displayed in Figure 2D. The prominent peaks were found at ≈529.4 and ≈531.7 eV and attributed to lattice oxygen (O^2–^) and oxygen vacancy (O_v_), respectively.^44,45,76^ The deconvoluted C 1s spectrum for g-CN in the composite is shown in Figure 2E. The pristine graphitic sites within the carbon nitride array (C–N–C) are associated with the peak at ≈284.6 eV; sp^2^-hybridized carbon atoms bound to N in an aromatic ring (N=C–N_2_) are associated with the peak at ≈285.7 eV; sp^2^-hybridized carbon in an aromatic group related to the C=N is associated with the peak at ≈288.7 eV. At binding energies of ≈397.5, ≈398.8, and 401.1 eV, three deconvolution peaks were also visible in the core level N 1s spectra (Figure 2F). These peaks were linked to sp^2^-bound aromatic in nitrogen to carbon atoms in the triazine structure, connecting tertiary nitrogen, terminal amino functional groups due to uncondensed thermal polymerization, and C=N^–^.^44,45^ Moreover, the XRD and XPS results verified the existence of metallic silver nickel oxide and carbon nitride in the produced samples.

### Surface Morphology Analysis

**Figure 3:**
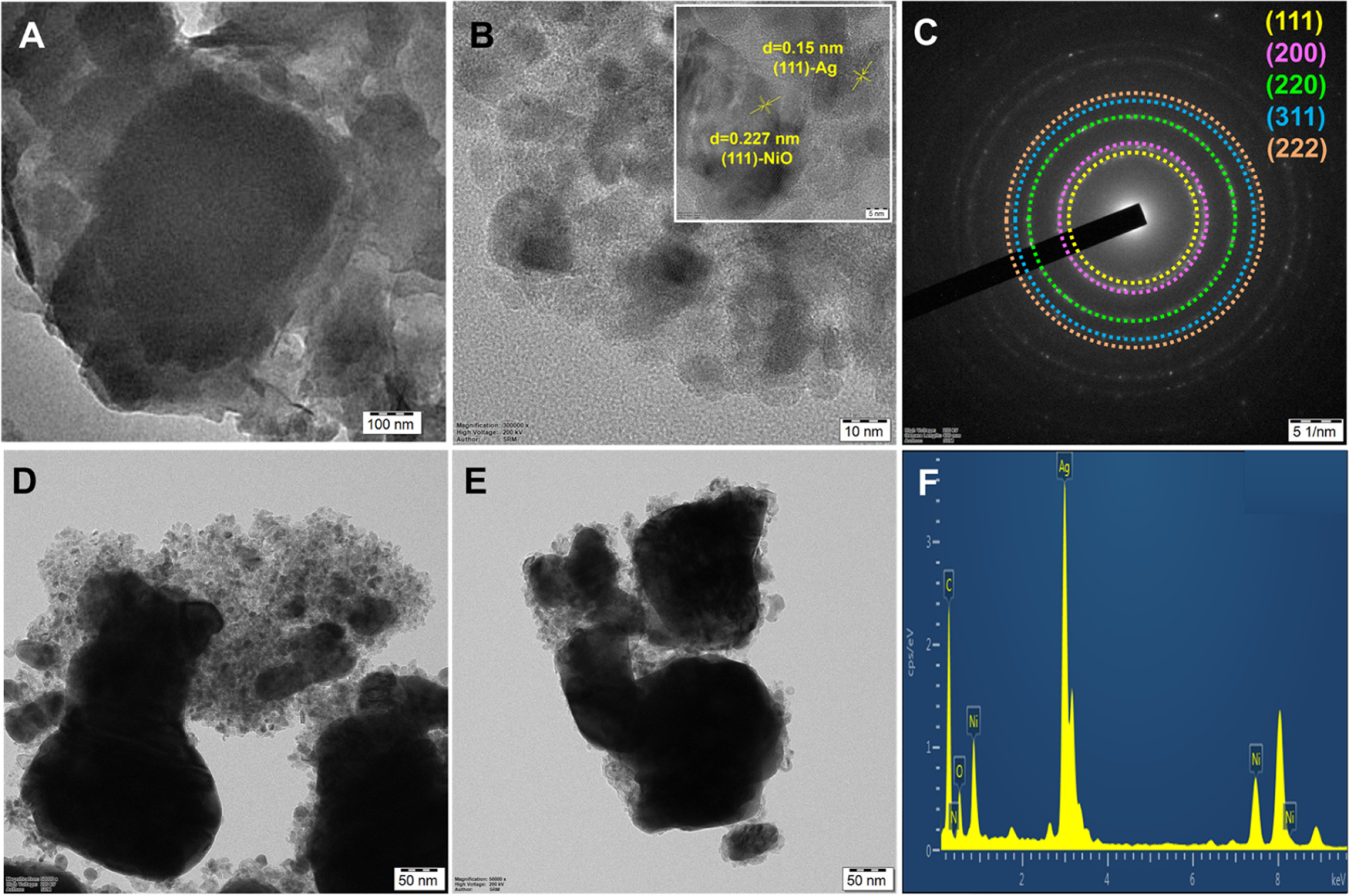
TEM images of (A) g-CN and (B) Ag/NiO nanoparticles with (B inset) its lattice fringes and (C) SAED patterns. (D, E) TEM images of Ag/NiO/g-CN composite and its EDX spectrum (F).

HR-SEM images of g-CN, Ag/NiO, and the Ag/NiO/g-CN composite are presented in Figure 3A–E. The microstructure of g-CN is layered, stacked, and crumpled, with a 2D lamellar pattern that has been structured (Figure 3A). The Ag/NiO nanoparticle nanoarrays were seen. Ag/NiO nanoarrays are shown in HR-TEM images in Figure 3B. This nanoarray particle is more practical for electrochemical sensor applications because of its higher active surface area and higher amount of active material in a unit area at the electrode/electrolyte interface. Ag/NiO nanoparticles are formed by a process that includes nucleation, hydrolysis, the Ostwald ripening mechanism, and precipitation . The following chemical reactions explain the proposed mechanism (eqs 4–6).
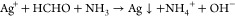
4
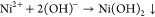
5
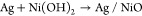
6

### Formation Mechanism

HMT split into formaldehyde and ammonia, and the ammonia then interacted to generate hydroxyl ions (OH^–^) and NH_4_^+^. Silver nitrate is reduced to metallic silver using formaldehyde as the reducing agent, and urea serves as the hydrolysis agent and breaks down in aqueous media to produce hydroxyl ions (OH^–^), which then combine with Ni^2+^ to generate Ni(OH)_2_. HMT regulates the supply of OH^–^ ions by controlling the rate of nucleation and hydrolysis.^77−79^ Numerous nuclei break down and develop into particles under the reaction circumstances, van der Waals forces, and crystal face attraction. The orientation of the crystallites is indicated by the measured interplanar spacing of *d* = 0.227 and 0.15 nm, which is consistent with *d*-spacing for the (111)-NiO and (111)-Ag planes shown in the XRD patterns (Figure 3B inset image). The crystalline definition and homogeneity of the nanoparticles can be recognized in these images. Moreover, circular rings corresponding to the (111), (200), (220), (311), and (222) planes showing enhanced crystalline nature of Ag/NiO are shown in the selected area electron diffraction (SAED) pattern in Figure 3C. Furthermore, the g-CN nanosheets comprising Ag/NiO nanoparticles anchored composite as shown in Figure 3D,E. EDS spectra analysis is used to analyze Ag/NiO/g-CN further, as illustrated in Figure 3F, which confirms the composite’s purity.

### Electrode Electrochemical Behavior in Redox Probe

**Figure 4:**
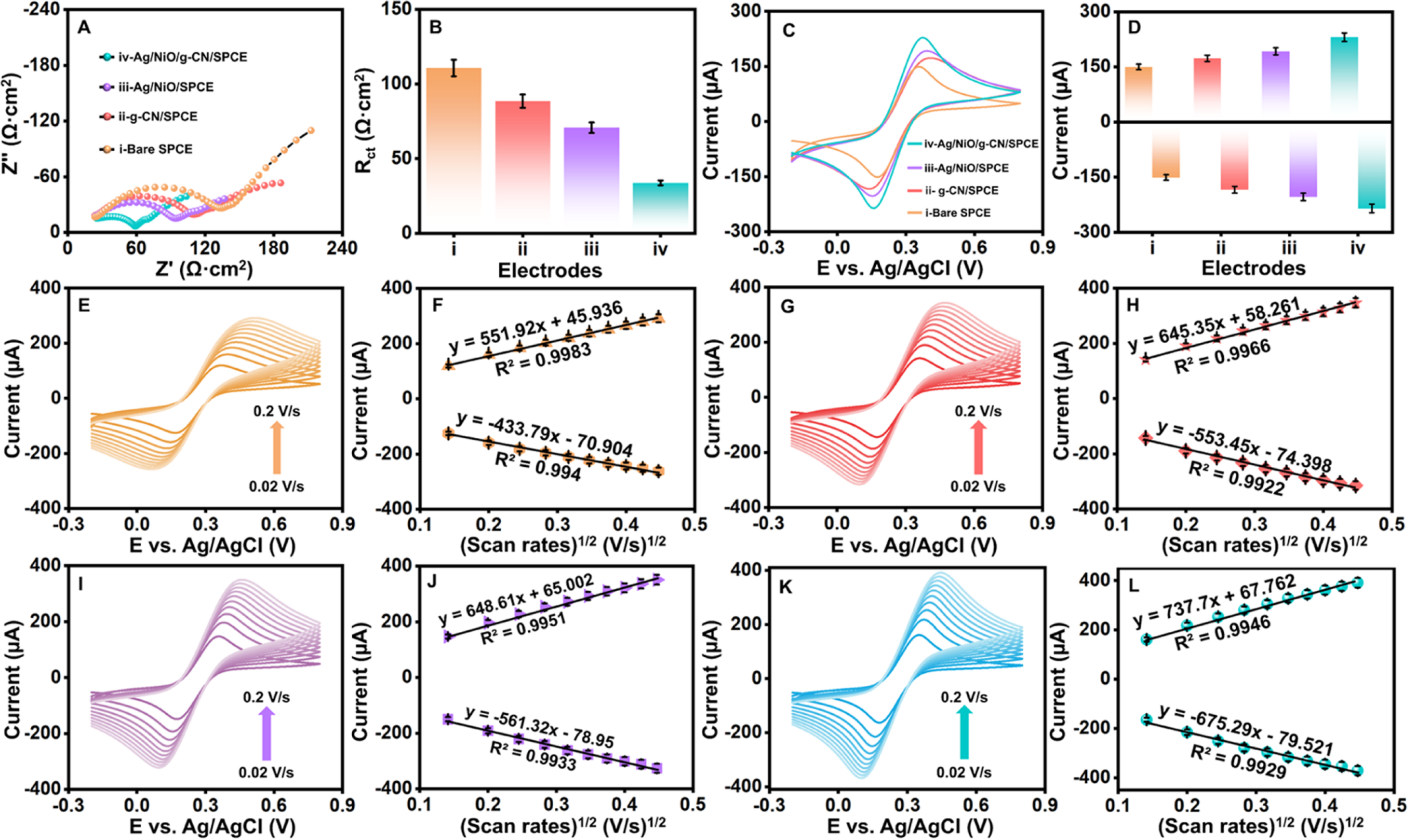
(A, B) EIS and (C, D) CVs of bare, g-CN, Ag/NiO, and Ag/NiO/g-CN-modified SPCEs in redox probe solution (5 mM [Fe(CN)_6_]^3**–**/4**–**^ and 0.1 M KCl). CVs of (E, F) bare SPCE, (G, H) g-CN/SPCE, (I, J) Ag/NiO/SPCE, and (K, L) Ag/NiO/g-CN/SPCE at different scan rates from 0.02 to 0.2 V/s in 5 mM [Fe(CN)_6_]^3**–**/4**–**^ and 0.1 M KCl with its corresponding linear plots, respectively.

The variations in the interfacial electrochemical characteristics of the electrode surface and the supporting electrolyte were explored using the electrochemical impedance spectroscopy (EIS) technique. EIS is a nondestructive electrochemical technique measuring differences in modified electrode surface characteristics. The following properties such as *R*_ct_, *R*_s_, *Z*_w_, and *C*_dl_ were obtained from the data analyzed from EIS fitted using Randle’s equivalent circuit, which stands for charge transfer resistance, solution resistance, Warburg impedance, and double-layer electron transfer resistance. In the higher frequency range of the EIS spectra, the electron transfer resistance (*R*_ct_) is shown as a semicircle, and at lower frequencies, electron diffusion is indicated by linearity. The Nyquist curves in 0.1 M KCl containing 5.0 mM [Fe(CN)_6_]^3**–**/4**–**^ for bare SPCE, g-CN/SPCE, Ag/NiO/SPCE, and Ag/NiO/g-CN modified SPCE (Figure 4A,B). The bare SPCE’s semicircle diameter is significant due to the unmodified electrode’s higher resistance, and its *R*_ct_ value was 110.69 Ω·cm^2^. The g-CN/SPCE electrode showing low resistance of *R*_ct_ 88.47 Ω showed how g-CN’s presence improved electron transport and offered a sizable active surface area. As Ag/NiO modified SPCE, the *R*_ct_ value decreased to 70.7 Ω·cm^2^, associated with Ag/NiO’s increased electrical conductivity. The *R*_ct_ value of the Ag/NiO/g-CN-modified SPCE drops to 33.71 Ω·cm^2^, which is remarkably lower than the *R*_ct_ values of the other modified and unmodified electrodes. The *R*_ct_ values are clearly plotted toward the electrodes for better comparison shown as a histogram graph in Figure 4B. The charge transfer coefficient (*K*_s_) rates for the g-CN/SPCE, Ag/NiO/SPCE, and Ag/NiO/g-CN/SPCE were calculated using the following formula (eq 7):
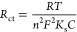
7where *K*_s_ is the charge transfer rate, *C* is the [Fe(CN)_6_]^4**–**/3**–**^ concentration, *n* is the total amount of electrons, and *R*, *T*, and *F* have their usual definitions. In comparison to other electrodes such as g-CN/SPCE (5.92 × 10^**–**7^ cm/s), and Ag/NiO/SPCE (7.4 × 10^**–**7^ cm/s), Ag/NiO/g-CN/SPCE exhibits outstanding electrocatalytic activity, with rate constants (*K*_s_) determined to be 1.55 × 10^**–**6^ cm/s. This suggests that electron transport between the electrode surface and the supporting electrolyte occurs rapidly. This is most likely due to the increased surface area, electron conductivity, and synergistic effect of the Ag/NiO and g-CN nanocomposite, which enable effective electrochemical detection of vanillin and speed up the electron transfer process on the surface of the Ag/NiO/g-CN-modified SPCE.

### Randles–Sevcik Equation (A)

The electron transferability at the bare SPCE, g-CN/SPCE, Ag/NiO/SPCE, and Ag/NiO/g-CN/SPCE is measured by cyclic voltammograms (CV) in a solution containing 0.1 M KCl and 5.0 mM [Fe(CN)_6_]^3–/4–^ at a scan rate of 50 mV/s at the potential between −0.2 and 0.6 V vs Ag/AgCl (Figure 4C,D). The modified electrode has prominent redox peaks because the [Fe(CN)_6_]^3–/4–^ redox pair is quasi-reversible. Table 2 presents a comparison of the measured potential differences (Δ*E*_p_), oxidation and reduction peak currents (*I*_pa_ and *I*_pc_), and anodic and cathodic peak potentials (*E*_pa_ and *E*_pc_). Comparing the Ag/NiO/g-CN/SPCE to the other modified and unmodified electrodes, the Ag/NiO/g-CN/SPCE showed lower *E*_p_ and more significant redox peak currents. The favorable electrostatic interactions between the [Fe(CN)_6_]^4**–**/3**–**^ ions and the Ag/NiO/g-CN surface may cause this. The EIS and CV profiles’ better surface properties and those of Ag/NiO/g-CN/SPCE correspond well. Efficiency is increased for Ag/NiO/g-CN/SPCE due to quicker ion diffusion across exposed active sites. Thus, basal spacing does not affect electrocatalysis activity. After analyzing the data mentioned above, it is clear that the synergistic interaction between the Ag/NiO and g-CN significantly improves the composite’s electrochemical performance, which is why it is utilized in subsequent electrochemical research.

**Table 2:** Electrochemical Analytical Outcomes from Redox Probe

electrode	*I*_pa_ (μA)	*E*_pa_ (V)	*I*_pc_ (μA)	*E*_pc_ (V)	Δ*E*_p_ (mV)	*A* (cm^2^)
g-CN	88.47	0.4	–184.26	0.14	260	0.174
Ag/NiO	70.47	0.38	–203.74	0.15	230	0.176
Ag/NiO/g-CN	33.71	0.36	–235.15	0.16	200	0.199

Using CV in a solution containing 0.1 M KCl and 5.0 mM [Fe(CN)_6_]^3–/4–^ at various sweep speeds (*v*), the electrochemically active surface area (A) of modified electrodes (Figure 4E–L**)**, such as (E,F) bare SPCE, (G,H) g-CN/SPCE, (I,J) Ag/NiO/SPCE, and (K,L) Ag/NiO/g-CN/SPCE, was calculated using the Randles–Sevcik equation (A) (eq 8). Furthermore, the Ag/NiO/g-CN/SPCE current increases when the scan rate increases. The square root graph of scan rate versus redox current is shown. Plotting the oxidation peak potential calibration plot against the natural logarithm of the scan rate was done.
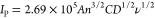
8where *C* is the [Fe(CN)_6_]^4**–**/3**–**^ electrolyte concentration in bulk solution (5 mM), *I*_p_ is the peak current, *A* is the modified electrode’s surface area, *n* is the number of electrons involved in the chemical reaction, *D* is the diffusion coefficient, and ν is the scan rate. The active surface area for g-CN/SPCE, Ag/NiO/SPCE, and Ag/NiO/g-CN/SPCE was determined to be 0.174, 0.176, and 0.199 cm^2^, respectively. The order of peak current intensity from low to high is bare SPCE < g-CN/SPCE< Ag/NiO/SPCE < Ag/NiO/g-CN/SPCE.

### Electrochemical Behaviors of Bare SPCE, g-CN/SPCE, Ag/NiO/SPCE, and Ag/NiO/g-CN/SPCE through the Detection of Vanillin

**Figure 5:**
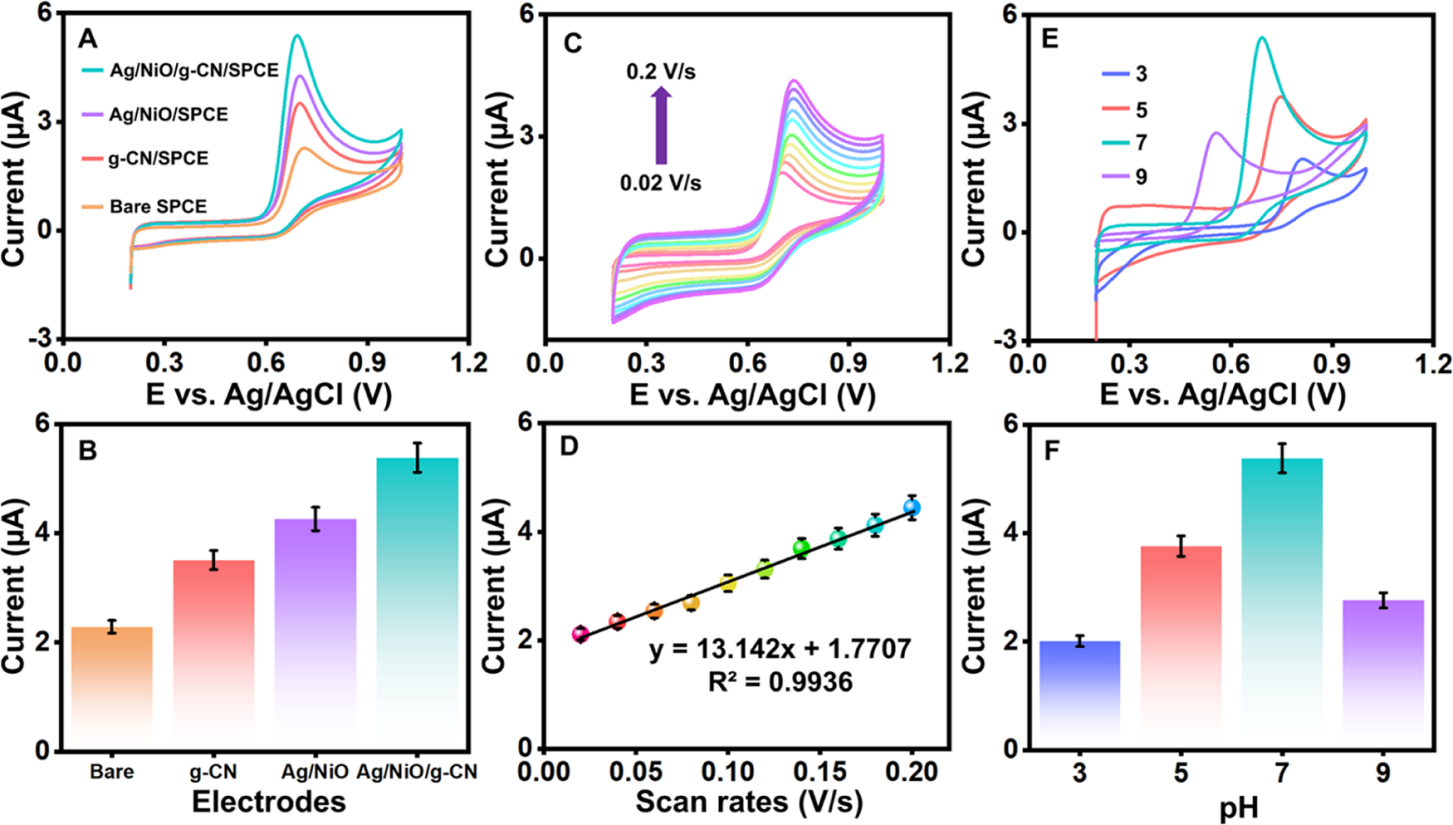
(A) CVs of bare, g-CN, Ag/NiO, and Ag/NiO/g-CN-modified SPCEs with vanillin and its bar diagram of electrodes versus current (μA) (B). (C) CVs of Ag/NiO/g-CN-modified SPCE with the increasing scan rates from 0.02 to 0.2 V/s and its corresponding linear plot of current (μA) versus scan rate (V/s) (D). (E) CVs of Ag/NiO/g-CN-modified SPCE at different supporting electrolytes (pH 3 to pH 9) and its corresponding linear plot of current (μA) versus pH (F).

The electrochemical performance and the variation of the current response of several catalysts of both modified and unmodified electrodes, such as bare SPCE, Ag/NiO/SPCE, g-CN/SPCE, and Ag/NiO/g-CN/SPCE, were studied at a scan rate of 50 mV/s in 0.1 M PB (pH 7) containing 100 μM vanillin (Figure 5A). The inadequate electron transport of the bare electrode shows a lower response toward vanillin. A prominent oxidation peak was seen on the bare SPCE at 0.71 V with a lower oxidation current (*I*_pa_) of 2.29 μA after adding 100 μM vanillin. This indicates that the charge transfer kinetics of the electrodes toward the oxidation of vanillin are not good. The bare SPCE was modified using g-C_3_N_4_ and Ag/NiO to detect vanillin. In this case, a noticeably smaller oxidation potential was recorded at 0.7 V, with a higher peak current of *I*_pa_ = 3.5 seen in the modification employing g-CN/SPCE. Compared with the bare electrode, it considerably improves vanillin oxidation at a potential (V) and current (μA). Likewise, by utilizing Ag/NiO to modify SPCE, improved electrochemical oxidation vanillin was demonstrated, exhibiting an oxidizing potential of 0.697 V and a higher current response of *I*_pa_= 4.26 μA. Remarkably, compared with all modified and unmodified electrodes, Ag/NiO/g-CN/SPCE exhibits a more notable oxidation current at around *I*_pa_ = 5.381 μA, with a peak potential of 0.69 V. Good sensitivity is achieved by having more catalytic sites in the improved electrode and analyte as clearly shown in Figure 5B. Higher electrocatalytic activity and the synergistic effect of Ag/NiO, g-CN, and π-stacking interaction among vanillin with Ag/NiO/g-CN are responsible for this exceptional performance. The vanillin’s oxidation potential and peak currents are shown on various modified electrodes. The calibration graph was created by connecting the electrodes to each vanillin’s current and reduction potential. Regarding the electro-oxidation of vanillin, the Ag/NiO/g-CN-modified SPCE has shown more excellent catalytic activity than any other modified electrode since it reduces at a lower overpotential.

### Effect of Scan Rate

The scan rate strongly influences the electrocatalytic behavior of the electrodes proposed for electrochemical sensors. CVs were scanned with the Ag/NiO/g-CN/SPCE nanocomposite, as shown, at 0.02–0.2 V/s with the presence of 50 μM vanillin (Figure 5C). This made it possible for peak potentials to gradually shift to the positive side, indicating the electro-oxidation of vanillin, which was probably caused by kinetic limits and mass transfer. The vanillin oxidation peak current varies linearly at the scan rate. This implies that the electrode surface has surface-controlled kinetics and that the surface shift to the positive side is irreversible. Additionally, Figure 5D shows the plot of the peak current vs the scan rate, as given in eq 9:
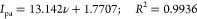
9for current *I*_pa_ in μA and scan rate ν in V/s. This clarifies that according to the vanillin reaction mechanism, the Ag/NiO/g-CN/SPCE follows a mixed (surface diffusion) regulated kinetics. A minor negative shift in the peak potential was observed when the scan rate increased to 200 mV/s.

### Effect of pH

The pH of the electrolytic solution significantly influences the redox behavior of electroactive species and molecules at the electrode interface. To evaluate the effect of pH on vanillin electro-oxidation, cyclic voltammetry (CV) was performed for 100 μM of vanillin in 0.1 M phosphate buffer (PB) over a pH range of 3–9 at a scan rate of 50 mV/s (Figure 5E).

The oxidation peak current and potential were found to vary with pH. At pH 5, the oxidation peak current was observed at 0.74 V with a significant current response of 3.76 μA, while at pH 3, the response appeared at a slightly higher potential of 0.8 V with a lower current of 2.01 μA. As the pH increased to 7, the oxidation potential shifted to 0.69 V, and the current response increased to 5.38 μA, indicating higher sensitivity at this pH. However, a further increase in pH to 9 resulted in a notable drop in current to 2.76 μA at 0.55 V, as shown in the bar diagram (Figure 5F).

The observed behavior can be explained based on the protonation and deprotonation of vanillin. The phenolic −OH group in vanillin has a p*K*_a_ of approximately 7. At pH values below the p*K*_a_ (acidic to near-neutral conditions), vanillin predominantly exists in its nondissociated (neutral) form. This neutral form exhibits a stronger adsorption affinity for the Ag/NiO/g-CN-modified SPCE surface, enhancing the oxidation current. Conversely, at pH values above the p*K*_a_ (basic conditions), vanillin exists primarily in its dissociated (deprotonated) form. The deprotonated form has weaker interactions with the electrode surface, likely due to electrostatic repulsion or reduced molecular adsorption, leading to a decrease in the oxidation current. While oxidation processes generally show greater sensitivity under basic conditions due to facilitated electron transfer, the results here emphasize that adsorption behavior plays a dominant role in determining the electrochemical response. The protonation state of vanillin significantly influences its interaction with the electrode surface, and acidic to near-neutral conditions are favorable for achieving higher current responses. Therefore, the electrochemical oxidation of vanillin on the Ag/NiO/g-CN-modified SPCE demonstrates pH-dependent behavior, with optimal current response observed at pH 7. The results highlight the importance of protons in the vanillin oxidation process, confirming the influence of protonation/deprotonation on both adsorption and electron transfer mechanisms.

### Effect of Vanillin Concentrations

**Figure 6:**
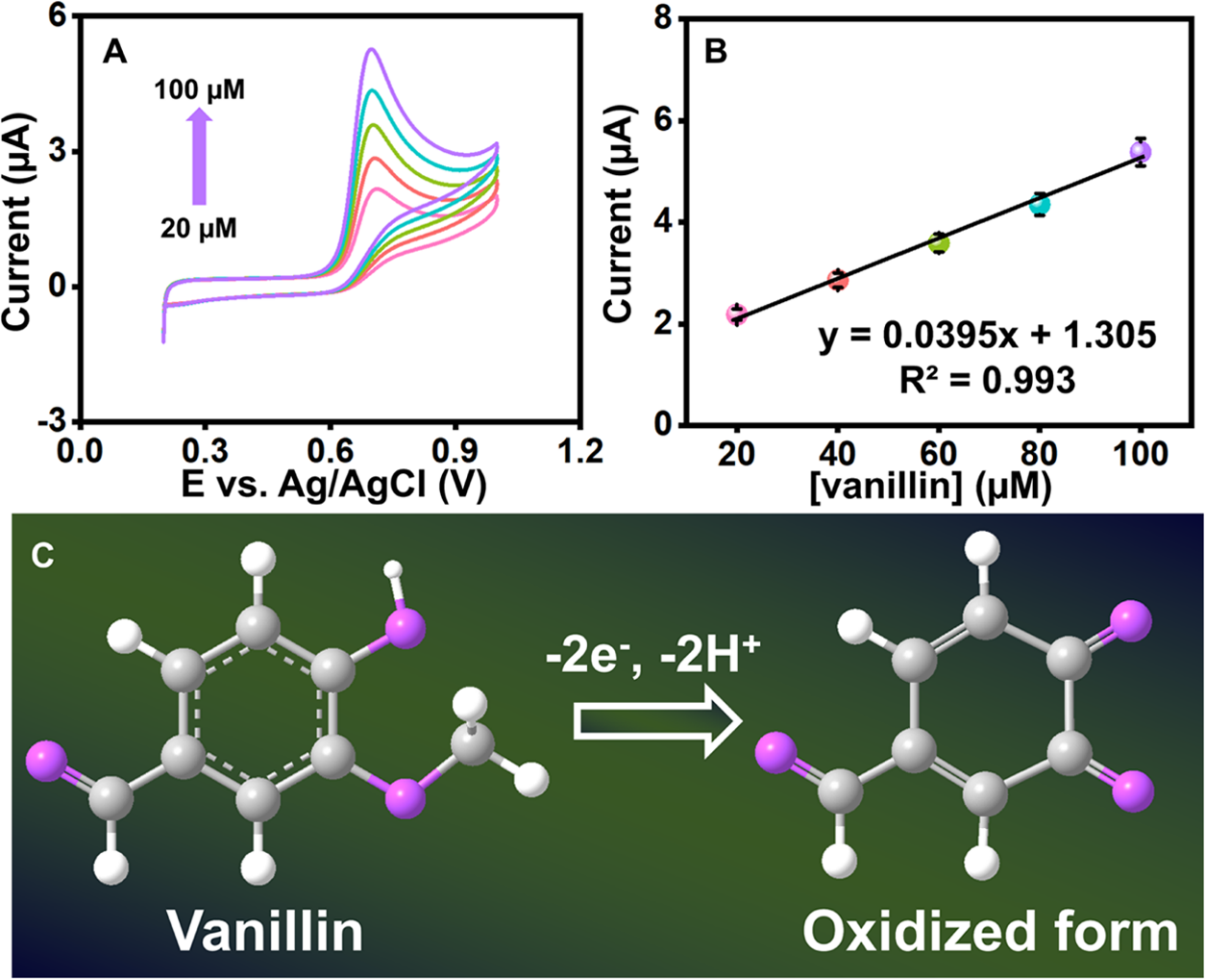
(A) CVs of Ag/NiO/g-CN-modified SPCE with the increasing concentration of vanillin from 20 to 100 μM and its corresponding linear plot of current (μA) versus concentration (μM) (B). (C) The overall possible vanillin oxidation mechanism.

The effect of concentration analysis of vanillin was then evaluated in 0.1 M PB (pH 7) utilizing Ag/NiO/g-CN/SPCE in the CV response (Figure 6A). When adding 20–100 μM, the anodic peak currents of the vanillin raised linearly. At the same time, the oxidation peak current was proportionate to the vanillin concentration, increasing the concentration of vanillin, and the current response also increased linearly. Saturation did not cause a decline in responses or a shift in the direction of the positive potential. These responses appropriately demonstrate the enhanced sensing capabilities of the electrode material. The current response vs vanillin concentration linear plot (Figure 6B) correlates with the linear regression (eq 10):
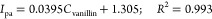
10for current *I*_pa_ in μA and vanillin concentration *C*_vanillin_ in μM.

In light of this, the Ag/NiO/g-CN-modified SPCE exhibits improved electro-reduction of vanillin detection and quick electron transfer. Further, the electrode surface was acquired by the overall possible vanillin oxidation mechanism shown in Figure 6C, displayed as 2e^**–**^ and 2H^+^ transfer.

### Sensor’s Analytical Performance

All of the optimization analyses for vanillin detection were optimized using the cyclic voltammetry technique. After that, the differential pulse voltammetry (DPV) method was used to analyze vanillin precisely at lower concentrations. Our proposed sensor system’s analytical performance features, such as the linear range of vanillin detection, the threshold of identification, and sensor sensitivities, were determined. The following equation (eq 11) calculates the limit of detection (LOD) of the proposed Ag/NiO/g-CN-modified SPCE toward vanillin:
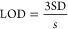
11where *s* is the calibration curve’s slope and SD is the standard deviations of the blank.

**Figure 7:**
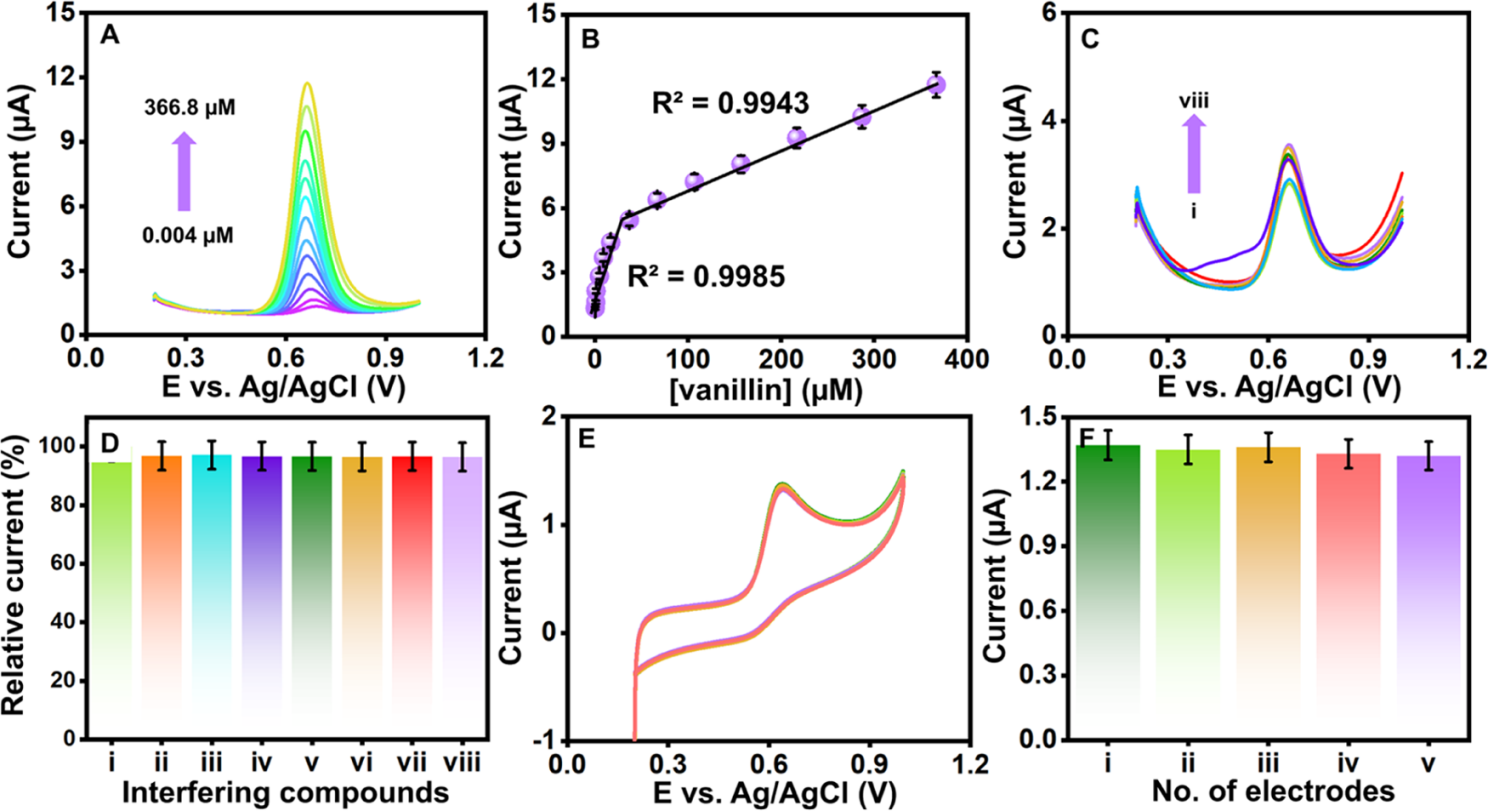
(A) DPVs of Ag/NiO/g-CN-modified SPCE with the increasing concentration of vanillin from 0.004 to 366.8 μM and its corresponding linear plot for concentration (μM) versus current (μA) (B). (C, D) Anti-interferent studies of Ag/NiO/g-CN-modified SPCE in vanillin’s presence and potential interfering compounds’ coexistence. (E, F) Reproducibility study of Ag/NiO/g-CN-modified SPCE in the presence of vanillin for five different packs of SPCEs.

The DPV analysis was achieved using Ag/NiO/g-CN-modified SPCE against different concentrations of vanillin from 0.004–366.8 μM in 0.1 M PB (∼7.0 pH). The current responses linearly increase with vanillin concentrations, as evidenced in Figure 7A. With increasing concentration, we see an increase in the response peaks. The remarkable sensing performance of the developed sensor was proved by the lack of a potential shift or the appearance of broader peaks at greater concentrations due to saturation. The calibration graph between *I*_pa_ and vanillin concentration with a correlation coefficient of *R*^2^ = 0.9985 (lower concentrations) and *R*^2^ = 0.9943 (higher concentration) is shown in Figure 7B. The calculated LOD is 0.9 nM from the wide linear ranges of 0.004–366.8 μM.

### Studies on Anti-Interferents, Reproducibility, and Stability

Selectivity of the sensor is crucial, and the rebinding procedure was conducted in the presence of common food adulterants, biomolecules, and other molecules, such as ii-caffeic acid (CA), iii-quercetin (QT), iv-*tert*-butylhydroquinone (TBHQ), v-theobromine (TB), vi-gallic acid (GA), vii-ferulic acid (FA), and viii-propyl gallate (PG) whose similar types/structure of i-vanillin molecules, to assess the selectivity of vanillin response (Figure 7C). The Ag/NiO/g-CN-modified SPCE demonstrated a higher sensitivity toward vanillin than other interfering molecules under investigation (Figure 7D). These results show that the Ag/NiO/g-CN-modified SPCE electrode surface preferentially recognizes the vanillin molecule in the presence of the interfering compounds.

The reproducibility of the fabricated sensor results in five times using the CV method is displayed in Figure 7E. In the initial current response of 20 μM vanillin in 0.1 M PB at pH 7.0, there were no discernible changes in the current responses or oxidation potential at Ag/NiO/g-CN-modified SPCE. The designed probe should function similarly at various electrode types. As demonstrated in Figure 7F, the bar diagram clearly shows the current versus different electrodes. However, there were slight variations in the anodic peak current with a relative standard deviation (RSD) of ±1.54%. These changes might be the outcome of the study’s surroundings having an impact.

Additionally, as shown in Figure S1, the sensor’s cyclic stability was verified using CV with 30 continuous cycles in vanillin’s presence. These alterations happened over several cycles as a result of environmental factors. The above electrochemical studies prove that our proposed sensor has excellent selectivity, reproducibility, and cyclic stability toward the electrochemical detection of vanillin.

### Real Sample Preparation

For the actual sample analysis, ice cream, chocolate, cookies, and cake were particularly selected. The investigation used commercial packets of each from Taipei supermarkets.

To melt the chocolate bar, about 0.5 g was added to 20 mL of deionized water and cooked at 60 °C in a water bath using the double-boiling method. The sample was then centrifuged at 4000*g* for 5 min to separate the supernatant, which was then diluted in 0.1 M PBS for further analysis and stored at 4 °C before being used. Similarly, all other actual samples are prepared using conventional protocols.

### Real-Time Applications

**Figure 8:**
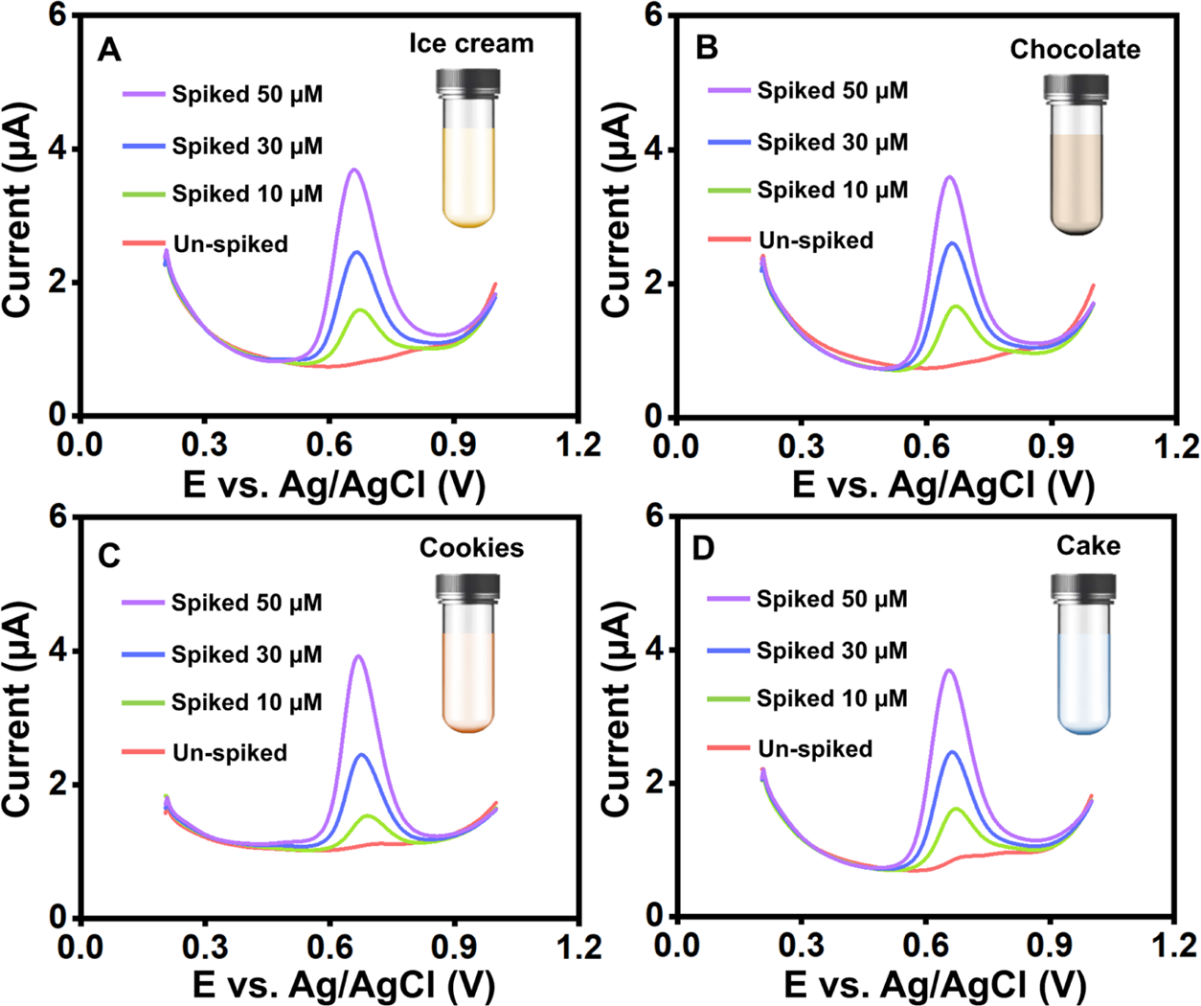
DPVs of Ag/NiO/g-CN-modified SPCE in spiked vanillin in (A) ice cream, (B) chocolate, (C) cookies, and (D) cake samples.

Real-world samples are being used to conduct an evaluation of the practical applicability of the sensor that has been provided. It is essential to validate the important uses of the recommended Ag/NiO/g-CN/SPCE detection (Figure 8A–D) to achieve correct vanillin concentration detection in samples of ice cream, chocolate, cookies, and cakes and to ensure that the detection is accurate. It worked well when the Ag/NiO/g-CN composite was tested for vanillin detection using real samples following pretreatment. The feasibility of the Ag/NiO/g-CN-modified sensor was evaluated using DPV analysis conducted on food samples. The standard addition method determines the vanillin concentration with good recovery rates. These findings suggest that Ag/NiO/g-CN/SPCE, whose recovery ranges are within reasonable bounds, can be helpful in vanillin monitoring in real-world samples.

## Conclusions

The significant threats that vanillin, a class of favoring agent most frequently used in foodstuffs, pose to the environment and public health have attracted much attention. Owing to this, the efficacy of their monitoring has arisen recently. We employed a vanillin detection by ultilizing Ag/NiO/g-CN-based electrochemical sensors. The Ag/NiO/g-CN nanohybrid was synthesized using a simple surfactant-assisted method, and their crystalline surface chemical properties were confirmed by XRD, FT-IR, XPS, and HR-TEM methods. The surface morphology analysis revealed that the nano-structured nanoarrays were self-assembled by synergistic interactions between Ag/NiO and g-CN nanosheets. The Ag/NiO/g-CN-modified SPCE identified vanillin more efficiently than other modified electrodes. The analytical applicability was analyzed using the i–t curve and DPV methods, demonstrating a more extended linear detection range from 0.004 to 366.8 μM with excellent LOD values of 0.9 nM. Our findings showed a significant interaction between Ag/NiO and g-CN, which may have increased electrocatalytic activity by encouraging electron transit inside vanillin and the enhanced SPCE surface. The sensor’s selectivity toward vanillin was examined when common interferents were present. The results demonstrate excellent selectivity, remarkable reproducibility, and higher stability. Numerous electrochemical investigations can also make use of the electrocatalyst. The developed Ag/NiO/g-CN sensor found vanillin with high recovery percentages in food samples.

## Supporting Information Available

Details of chemicals and reagents; details of instrumentation and methods; cyclic stability of Ag/NiO/g-CN; comparison table of Ag/NiO/g-CN/SPCE toward vanillin detection (PDF)

## References

[ref1] Ning J.; He Q.; Luo X.; Wang M.; Liu D.; Wang J.; Liu J.; Li G. Rapid and sensitive determination of vanillin based on a glassy carbon electrode modified with Cu_2_O-electrochemically reduced graphene oxide nanocomposite film. Sensors 2018, 18 (9), 2762 10.3390/s18092762. 30135387 PMC6164793

[ref2] Pushpanjali P.A.; Manjunatha J.G.; Tigari G.; Fattepur S. Poly (niacin) based carbon nanotube sensor for the sensitive and selective voltammetric detection of vanillin with caffeine. Anal. Bioanal. Electrochem. 2020, 12 (4), 553–568.

[ref3] Shoeb A.; Chowta M.; Pallempati G.; Rai A.; Singh A. J. Evaluation of antidepressant activity of vanillin in mice. Indian J. Pharmacol. 2013, 45 (2), 141–144. 10.4103/0253-7613.108292. 23716889 PMC3660925

[ref4] Kim M. E.; Na J. Y.; Park Y.-D.; Lee J. S. Anti-neuroinflammatory effects of vanillin through the regulation of inflammatory factors and NF-κB signaling in LPS-stimulated microglia. Appl. Biochem. Biotechnol. 2019, 187, 884–893. 10.1007/s12010-018-2857-5. 30097802

[ref5] Deng P.; Xu Z.; Zeng R.; Ding C. Electrochemical behavior and voltammetric determination of vanillin based on an acetylene black paste electrode modified with graphene–polyvinylpyrrolidone composite film. Food Chem. 2015, 180, 156–163. 10.1016/j.foodchem.2015.02.035. 25766813

[ref6] Srinual S.; Chanvorachote P.; Pongrakhananon V. J. Suppression of cancer stem-like phenotypes in NCI-H460 lung cancer cells by vanillin through an Akt-dependent pathway. Int. J. Oncol. 2017, 50 (4), 1341–1351. 10.3892/ijo.2017.3879. 28259926

[ref7] Mourtzinos I.; Konteles S.; Kalogeropoulos N.; Karathanos V. T. Thermal oxidation of vanillin affects its antioxidant and antimicrobial properties. Food Chem. 2009, 114 (3), 791–797. 10.1016/j.foodchem.2008.10.014.

[ref8] Jiang L.; Ding Y.; Jiang F.; Li L.; Mo F. Electrodeposited nitrogen-doped graphene/carbon nanotubes nanocomposite as enhancer for simultaneous and sensitive voltammetric determination of caffeine and vanillin. Anal. Chim. Acta 2014, 833, 22–28. 10.1016/j.aca.2014.05.010. 24909770

[ref9] Dong Z.; Gu F.; Xu F.; Wang Q. Comparison of four kinds of extraction techniques and kinetics of microwave-assisted extraction of vanillin from Vanilla planifolia Andrews. Food Chem. 2014, 149, 54–61. 10.1016/j.foodchem.2013.10.052. 24295676

[ref10] Lee Y.-Y.; Sriram B.; Wang S.-F.; Stanley M. M.; Lin W.-C.; Kogularasu S.; Chang-Chien G.-P.; George M. Eco-innovative electrochemical sensing for precise detection of vanillin and sulfadiazine additives in confectioneries. Appl. Surf. Sci. Adv. 2024, 20, 100584 10.1016/j.apsadv.2024.100584.

[ref11] Arun R.; Arafat A. S. S. Y.; D’Souza C. J.; Sivaramakrishnan V.; Dhananjaya B. L. Vanillin Analog–Vanillyl Mandelic Acid, a Novel Specific Inhibitor of Snake Venom 5′-Nucleotidase. Arch. Pharm. 2014, 347 (9), 616–623. 10.1002/ardp.201400069. 25042467

[ref12] Alpar N.; Yardım Y.; Şentürk Z. Selective and simultaneous determination of total chlorogenic acids, vanillin and caffeine in foods and beverages by adsorptive stripping voltammetry using a cathodically pretreated boron-doped diamond electrode. Sens. Actuators, B 2018, 257, 398–408. 10.1016/j.snb.2017.10.100.

[ref13] Chen L.; Chaisiwamongkhol K.; Chen Y.; Compton R. G. Rapid Electrochemical Detection of Vanillin in Natural Vanilla. Electroanalysis 2019, 31 (6), 1067–1074. 10.1002/elan.201900037.

[ref14] Erady V.; Mascarenhas R. J.; Satpati A. K. Highly efficient and selective quantification of vanillin in food, beverages and pharmaceuticals using surfactant modified carbon paste sensor. Sens. Int. 2020, 1, 100023 10.1016/j.sintl.2020.100023.

[ref15] Beitollahi H.; Khalilzadeh M. A.; Tajik S.; Safaei M.; Zhang K.; Jang H. W.; Shokouhimehr M. Recent advances in applications of voltammetric sensors modified with ferrocene and its derivatives. ACS Omega 2020, 5 (5), 2049–2059. 10.1021/acsomega.9b03788. 32064365 PMC7016907

[ref16] Cheraghi S.; Taher M. A.; Karimi-Maleh H. J. Highly sensitive square wave voltammetric sensor employing CdO/SWCNTs and room temperature ionic liquid for analysis of vanillin and folic acid in food samples. J. Food Compos. Anal. 2017, 62, 254–259. 10.1016/j.jfca.2017.06.006.

[ref17] Cho I.-H.; Kim D. H.; Park S. Electrochemical biosensors: perspective on functional nanomaterials for on-site analysis. Biomater. Res. 2020, 24 (1), 6 10.1186/s40824-019-0181-y. 32042441 PMC7001310

[ref18] Choi E. J.; Bae S. H.; Park J. B.; Kwon M. J.; Jang S. M.; Zheng Y. F.; Lee Y. S.; Lee S.-J.; Bae S. K. Simultaneous quantification of caffeine and its three primary metabolites in rat plasma by liquid chromatography–tandem mass spectrometry. Food Chem. 2013, 141 (3), 2735–2742. 10.1016/j.foodchem.2013.05.069. 23871018

[ref19] Curulli A. Electrochemical Biosensors in Food Safety: Challenges and Perspectives. Molecules 2021, 26 (10), 2940 10.3390/molecules26102940. 34063344 PMC8156954

[ref20] Fu L.; Xie K.; Wu D.; Wang A.; Zhang H.; Ji Z. Electrochemical determination of vanillin in food samples by using pyrolyzed graphitic carbon nitride. Mater. Chem. Phys. 2020, 242, 122462 10.1016/j.matchemphys.2019.122462.

[ref21] Haššo M.; Matúšková I.; Švorc L’. Easy, rapid and high-throughput analytical sensing platform for theobromine quantification in chocolate and cocoa products based on batch injection analysis with amperometric detection. J. Food Compos. Anal. 2023, 115, 105035 10.1016/j.jfca.2022.105035.

[ref22] Huang L.; Hou K.; Jia X.; Pan H.; Du M. Preparation of novel silver nanoplates/graphene composite and their application in vanillin electrochemical detection. Mater. Sci. Eng. 2014, 38, 39–45. 10.1016/j.msec.2014.01.037. 24656350

[ref23] López R.; Aznar M.; Cacho J.; Ferreira V. J. Determination of minor and trace volatile compounds in wine by solid-phase extraction and gas chromatography with mass spectrometric detection. J. Chromatogr. A 2002, 966 (1–2), 167–177. 10.1016/S0021-9673(02)00696-9. 12214691

[ref24] Pérez-Esteve É.; Lerma-García M. J.; Fuentes A.; Palomares C.; Barat J. M. Control of undeclared flavoring of cocoa powders by the determination of vanillin and ethyl vanillin by HPLC. Food Control 2016, 67, 171–176. 10.1016/j.foodcont.2016.02.048.

[ref25] Shu M.; Man Y.; Ma H.; Luan F.; Liu H.; Gao Y. Determination of vanillin in milk powder by capillary electrophoresis combined with dispersive liquid-liquid microextraction. Food Anal. Methods 2016, 9, 1706–1712. 10.1007/s12161-015-0347-8.

[ref26] Timotheou-Potamia M.; Calokerinos A. C. Chemiluminometric determination of vanillin in commercial vanillin products. Talanta 2007, 71 (1), 208–212. 10.1016/j.talanta.2006.03.046. 19071290

[ref27] Peng H.; Wang S.; Zhang Z.; Xiong H.; Li J.; Chen L.; Li Y. J. Molecularly imprinted photonic hydrogels as colorimetric sensors for rapid and label-free detection of vanillin. J. Agric. Food Chem. 2012, 60 (8), 1921–1928. 10.1021/jf204736p. 22292481

[ref28] Raril C.; Manjunatha J. A simple approach for the electrochemical determination of vanillin at ionic surfactant modified graphene paste electrode. Microchem. J. 2020, 154, 104575 10.1016/j.microc.2019.104575.

[ref29] Yardım Y.; Gülcan M.; Şentürk Z. Determination of vanillin in commercial food product by adsorptive stripping voltammetry using a boron-doped diamond electrode. Food Chem. 2013, 141 (3), 1821–1827. 10.1016/j.foodchem.2013.04.085. 23870896

[ref30] Peng J.; Hou C.; Hu X. J. I. A graphene-based electrochemical sensor for sensitive detection of vanillin. Int. J. Electrochem. Sci. 2012, 7 (2), 1724–1733. 10.1016/S1452-3981(23)13447-X.

[ref31] ULUBAY KARABİBEROĞLU Ş.; KOÇAK Ç. C. Voltammetric determination of vanillin in commercial food products using overoxidized poly (pyrrole) film-modified glassy carbon electrodes. Turk. J. Chem. 2018, 42 (2), 291–305. 10.3906/kim-1704-21.

[ref32] Luo S.; Liu Y. Poly (acid chrome blue K) modified glassy carbon electrode for the determination of vanillin. Int. J. Electrochem. Sci. 2012, 7 (7), 6396–6405. 10.1016/S1452-3981(23)19489-2.

[ref33] Ghoreishi S. M.; Behpour M.; Khoobi A.; Salavati-Niasari M. Electrochemical study of a self-assembled monolayer of N, N′-bis [(E)-(1-pyridyl) methylidene]-1, 3-propanediamine formed on glassy carbon electrode: preparation, characterization and application. Anal. Methods 2013, 5 (23), 6727–6733. 10.1039/c3ay41480a.

[ref34] Darabi R.; Shabani-Nooshabadi M.; Khoobi A. A potential strategy for simultaneous determination of deferoxamine and vitamin C using MCR-ALS with nanostructured electrochemical sensor in serum and urine of thalassemia and diabetic patients. J. Electrochem. Soc. 2021, 168 (4), 046514 10.1149/1945-7111/abf6ed.

[ref35] Ghoreishi S. M.; Khoobi A.; Behpour M.; Masoum S. Application of multivariate curve resolution alternating least squares to biomedical analysis using electrochemical techniques at a nanostructure-based modified sensor. Electrochim. Acta 2014, 130, 271–278. 10.1016/j.electacta.2014.03.016.

[ref36] Khoobi A.; Soltani N.; Aghaei M. Computational design and multivariate statistical analysis for electrochemical sensing platform of iron oxide nanoparticles in sensitive detection of anti-inflammatory drug diclofenac in biological fluids. J. Alloys Compd. 2020, 831, 154715 10.1016/j.jallcom.2020.154715.

[ref37] Valian M.; Khoobi A.; Salavati-Niasari M. Synthesis, characterization and electrochemical sensors application of Tb_2_Ti_2_O_7_ nanoparticle modified carbon paste electrode for the sensing of mefenamic acid drug in biological samples and pharmaceutical industry wastewater. Talanta 2022, 247, 123593 10.1016/j.talanta.2022.123593. 35636361

[ref38] Khoobi A.; Ghoreishi S. M.; Masoum S.; Behpour M. Multivariate curve resolution-alternating least squares assisted by voltammetry for simultaneous determination of betaxolol and atenolol using carbon nanotube paste electrode. Bioelectrochemistry 2013, 94, 100–107. 10.1016/j.bioelechem.2013.04.002. 23632433

[ref39] Yarahmadi S.; Azadbakht A.; Derikvand R. M. Hybrid synthetic receptor composed of molecularly imprinted polydopamine and aptamers for impedimetric biosensing of urea. Microchim. Acta 2019, 186, 71 10.1007/s00604-018-3180-0. 30627876

[ref40] Roushani M.; Kohzadi S.; Haghjoo S.; Azadbakht A. Dual detection of Malation and Hg (II) by fluorescence switching of graphene quantum dots. Environ. Nanotechnol., Monit. Manage. 2018, 10, 308–313. 10.1016/j.enmm.2018.08.002.

[ref41] Gholivand M. B.; Azadbakht A. Fabrication of a highly sensitive glucose electrochemical sensor based on immobilization of Ni (II)–pyromellitic acid and bimetallic Au–Pt inorganic–organic hybrid nanocomposite onto carbon nanotube modified glassy carbon electrode. Electrochim. Acta 2012, 76, 300–311. 10.1016/j.electacta.2012.05.037.

[ref42] Gholivand M. B.; Azadbakht A.; Pashabadi A. Simultaneous Determination of Trace Zinc and Cadmium by Anodic Stripping Voltammetry Using a Polymeric Film Nanoparticle Self-Assembled Electrode. Electroanalysis 2011, 23 (2), 364–370. 10.1002/elan.201000395.

[ref43] Azadbakht A.; Derikvandi Z. Aptamer-based sensor for diclofenac quantification using carbon nanotubes and graphene oxide decorated with magnetic nanomaterials. J. Iran. Chem. Soc. 2018, 15 (3), 595–606. 10.1007/s13738-017-1259-x.

[ref44] Govindaraj M.; Rajendran J.; PK U. G.; Muthukumaran M. K.; Jayaraman B.; et al. Graphitic carbon nitride nanosheets decorated with strontium tungstate nanospheres as an electrochemical transducer for sulfamethazine sensing. ACS Appl. Nano Mater. 2023, 6 (2), 930–945. 10.1021/acsanm.2c04322.

[ref45] Govindaraj M.; PK U. G.; Muthukumaran M. K.; Sekar K.; Maruthapillai A.; Arockia Selvi J. Electrostatic Self Assembly of Metal-Free Hexagonal Boron Nitride/Protonated Carbon Nitride (h-BN/PCN) Nanohybrid: A Synergistically Upgraded 2D/2D Sustainable Electrocatalyst for Sulfamethazine Identification. ChemNanoMat 2023, 9 (11), e202300330 10.1002/cnma.202300330.

[ref46] Muthukumaran M. K.; Govindaraj M.; Raja B. K.; Arockia Selvi J. Crystal plane-integrated strontium oxide/hexagonal boron nitride nanohybrids for rapid electrochemical sensing of anticancer drugs in human blood serum samples. Anal. Methods 2023, 15 (42), 5639–5654. 10.1039/D3AY01493B. 37855090

[ref47] Muthukumaran M. K.; Govindaraj M.; Raja B. K.; Arockia Selvi J. In situ synthesis of polythiophene encapsulated 2D hexagonal boron nitride nanocomposite based electrochemical transducer for detection of 5-fluorouracil with high selectivity. RSC Adv. 2023, 13 (5), 2780–2794. 10.1039/D2RA07147A. 36756436 PMC9850362

[ref48] Raja B. K.; Govindaraj M.; Muthukumaran M. K.; Ramar S.; Arockia Selvi J. Activity augmentation of a functionalized 2D conjugated polymer matrix with iron vanadate nano-bulbs for real-time detection of levofloxacin. New J. Chem. 2024, 48 (35), 15324–15337. 10.1039/D4NJ02548B.

[ref49] Govindaraj M.; Srivastava A.; Muthukumaran M. K.; Tsai P.-C.; Lin Y.-C.; Raja B. K.; Rajendran J.; Ponnusamy V. K.; Arockia Selvi J. Current advancements and prospects of enzymatic and non-enzymatic electrochemical glucose sensors. Int. J. Biol. Macromol. 2023, 253, 126680 10.1016/j.ijbiomac.2023.126680. 37673151

[ref50] Shang L.; Zhao F.; Zeng B. J. F. c. Sensitive voltammetric determination of vanillin with an AuPd nanoparticles– graphene composite modified electrode. Food Chem. 2014, 151, 53–57. 10.1016/j.foodchem.2013.11.044. 24423501

[ref51] Gan T.; Shi Z.; Deng Y.; Sun J.; Wang H. J. E. A. Morphology–dependent electrochemical sensing properties of manganese dioxide–graphene oxide hybrid for guaiacol and vanillin. Electrochim. Acta 2014, 147, 157–166. 10.1016/j.electacta.2014.09.116.

[ref52] Deng P.; Xu Z.; Zeng R.; Ding C. Electrochemical behavior and voltammetric determination of vanillin based on an acetylene black paste electrode modified with graphene-polyvinylpyrrolidone composite film. Food Chem. 2015, 180, 156–163. 10.1016/j.foodchem.2015.02.035. 25766813

[ref53] Qianwen M.; Yaping D.; Li L.; Anqing W.; Dingding D.; Yijun Z. J. Electrospun MoS_2_ composite carbon nanofibers for determination of vanillin. J. Electroanal. Chem. 2019, 833, 297–303. 10.1016/j.jelechem.2018.09.040.

[ref54] Li J.; Feng H.; Li J.; Jiang J.; Feng Y.; He L.; Qian D. Bimetallic Ag-Pd nanoparticles-decorated graphene oxide: a fascinating three-dimensional nanohybrid as an efficient electrochemical sensing platform for vanillin determination. Electrochim. Acta 2015, 176, 827–835. 10.1016/j.electacta.2015.07.091.

[ref55] Liu Y.; Liang Y.; Lian H.; Zhang C.; Peng J. J. Sensitive Voltammetric determination of vanillin with an electrolytic manganese dioxide– graphene composite modified electrode. Int. J. Electrochem. Sci. 2015, 10 (5), 4129–4137. 10.1016/S1452-3981(23)06608-7.

[ref56] Lv T.; Yao Y.; Li N.; Chen T. Highly stretchable supercapacitors based on aligned carbon nanotube/molybdenum disulfide composites. Angew. Chem., Int. Ed. 2016, 55 (32), 9191–9195. 10.1002/anie.201603356. 27328623

[ref57] Liu C.; Yu Z.; Neff D.; Zhamu A.; Jang B. Z. Graphene-based supercapacitor with an ultrahigh energy density. Nano Lett. 2010, 10 (12), 4863–4868. 10.1021/nl102661q. 21058713

[ref58] Vijayakumar S.; Nagamuthu S.; Muralidharan G. Porous NiO/C nanocomposites as electrode material for electrochemical supercapacitors. ACS Sustainable Chem. Eng. 2013, 1 (9), 1110–1118. 10.1021/sc400152r.

[ref59] Lee Y.-Y.; Sriram B.; Wang S.-F.; Kogularasu S.; Chang-Chien G.-P. A comprehensive review on emerging role of rare earth oxides in electrochemical biosensors. Microchem. J. 2023, 193, 109140 10.1016/j.microc.2023.109140.

[ref60] Joseph X. B.; Sriram B.; Wang S.-F.; Baby J. N.; Hsu Y.-F.; George M. Revealing the effect of multidimensional ZnO@CNTs/RGO composite for enhanced electrochemical detection of flufenamic acid. Microchem. J. 2021, 168, 106448 10.1016/j.microc.2021.106448.

[ref61] Sriram B.; Baby J. N.; Wang S.-F.; George M.; Joseph X. B.; Tsai J.-T. Surface engineering of three-dimensional-like hybrid AB_2_O_4_ (AB= Zn, Co, and Mn) wrapped on sulfur-doped reduced Graphene oxide: investigation of the role of an Electrocatalyst for Clioquinol detection. ACS Appl. Electron. Mater. 2021, 3 (1), 362–372. 10.1021/acsaelm.0c00906.

[ref62] Nagamuthu S.; Vijayakumar S.; Ryu K.-S. Synthesis of Ag anchored Ag3VO4 stacked nanosheets: toward a negative electrode material for high-performance asymmetric supercapacitor devices. J. Phys. Chem. C 2016, 120 (34), 18963–18970. 10.1021/acs.jpcc.6b04925.

[ref63] Wu B.; Sheng M.; Gao S.; Wang Y.; Liao F. J. Single-source precursor to Ag/NiO composite for rechargeable charge storage. J. Alloys Compd. 2017, 692, 34–39. 10.1016/j.jallcom.2016.08.315.

[ref64] Júnior P. C. G.; dos Santos V. B.; Lopes A. S.; de Souza J. P. I.; Pina J. R. S.; Júnior G. C. A. C.; Marinho P. S. B. Determination of theobromine and caffeine in fermented and unfermented Amazonian cocoa (Theobroma cacao L.) beans using square wave voltammetry after chromatographic separation. Food Control 2020, 108, 106887 10.1016/j.foodcont.2019.106887.

[ref65] Kogularasu S.; Sriram B.; Wang S.-F.; Sheu J.-K. Sea-urchin-like Bi_2_S_3_ microstructures decorated with graphitic carbon nitride nanosheets for use in food preservation. ACS Appl. Nano Mater. 2022, 5 (2), 2375–2384. 10.1021/acsanm.1c04055.

[ref66] Sriram B.; Baby J.; Wang S.-F.; Ranjitha M.R.; Govindasamy M.; George M. Eutectic solvent-mediated synthesis of NiFe-LDH/sulfur-doped carbon nitride arrays: investigation of electrocatalytic activity for the dimetridazole sensor in human sustenance. ACS Sustainable Chem. Eng. 2020, 8 (48), 17772–17782. 10.1021/acssuschemeng.0c06070.

[ref67] Sriram B.; Baby J. N.; Hsu Y.-F.; Wang S.-F.; George M.; Veerakumar P.; Lin K.-C. Electrochemical sensor-based barium zirconate on sulphur-doped graphitic carbon nitride for the simultaneous determination of nitrofurantoin (antibacterial agent) and nilutamide (anticancer drug). J. Electroanal. Chem. 2021, 901, 115782 10.1016/j.jelechem.2021.115782.

[ref68] Sriram B.; Baby J. N.; Wang S.-F.; Govindasamy M.; George M.; Jothiramalingam R. Cobalt molybdate nanorods decorated on boron-doped graphitic carbon nitride sheets for electrochemical sensing of furazolidone. Microchim. Acta 2020, 187, 654 10.1007/s00604-020-04590-3. 33179119

[ref69] Sriram B.; Baby J. N.; Wang S.-F.; Hsu Y.-F.; Sherlin V. A.; George M. Well-designed construction of yttrium orthovanadate confined on graphitic carbon nitride sheets: electrochemical investigation of dimetridazole. Inorg. Chem. 2021, 60 (17), 13150–13160. 10.1021/acs.inorgchem.1c01548. 34428891

[ref70] Sriram B.; Baby J. N.; Hsu Y.-F.; Wang S.-F.; George M. Zirconium phosphate supported on g-C3N4 nanocomposite for sensitive detection of nitrite. J. Electrochem. Soc. 2021, 168 (8), 087502 10.1149/1945-7111/ac1707.

[ref71] Sriram B.; Gouthaman S.; Wang S.-F.; Hsu Y.-F. Cobalt molybdate hollow spheres decorated graphitic carbon nitride sheets for electrochemical sensing of dimetridazole. Food Chem. 2024, 430, 136853 10.1016/j.foodchem.2023.136853. 37541041

[ref72] Sriram B.; Baby J. N.; Hsu Y.-F.; Wang S.-F.; Benadict Joseph X.; George M.; Veerakumar P.; Lin K. C. MnCo_2_O_4_ microflowers anchored on P-doped g-C_3_N_4_ nanosheets as an electrocatalyst for voltammetric determination of the antibiotic drug sulfadiazine. ACS Appl. Electron. Mater. 2021, 3 (9), 3915–3926. 10.1021/acsaelm.1c00506.

[ref73] Nagamuthu S.; Vijayakumar S.; Lee S.-H.; Ryu K.-S. Hybrid supercapacitor devices based on MnCo_2_O_4_ as the positive electrode and FeMn_2_O_4_ as the negative electrode. Appl. Surf. Sci. 2016, 390, 202–208. 10.1016/j.apsusc.2016.08.072.

[ref74] Lu W.; Liu G.; Gao S.; Xing S.; Wang J. Tyrosine-assisted preparation of Ag/ZnO nanocomposites with enhanced photocatalytic performance and synergistic antibacterial activities. Nanotechnology 2008, 19 (44), 445711 10.1088/0957-4484/19/44/445711. 21832753

[ref75] Nithya V.; Selvan R. K.; Vasylechko L. J. Hexamethylenetetramine assisted hydrothermal synthesis of BiPO4 and its electrochemical properties for supercapacitors. J. Phys. Chem. Solids 2015, 86, 11–18. 10.1016/j.jpcs.2015.06.007.

[ref76] Ren Y.; Gao L. J. From three-dimensional flower-like α-Ni(OH)_2_ nanostructures to hierarchical porous NiO nanoflowers: microwave-assisted fabrication and supercapacitor properties. J. Am. Ceram. Soc. 2010, 93 (11), 3560–3564. 10.1111/j.1551-2916.2010.04090.x.

[ref77] Davar F.; Fereshteh Z.; Salavati-Niasari M. J. Nanoparticles Ni and NiO: synthesis, characterization and magnetic properties. J. Alloys Compd. 2009, 476 (1–2), 797–801. 10.1016/j.jallcom.2008.09.121.

[ref78] Barakat A.; Al-Noaimi M.; Suleiman M.; Aldwayyan A. S.; Hammouti B.; Ben Hadda T.; Haddad S. F.; Boshaala A.; Warad I. One step synthesis of NiO nanoparticles via solid-state thermal decomposition at low-temperature of novel aqua (2, 9-dimethyl-1, 10-phenanthroline) NiCl_2_ complex. Int. J. Mol. Sci. 2013, 14 (12), 23941–23954. 10.3390/ijms141223941. 24351867 PMC3876087

[ref79] Peck M. A.; Langell M. A. Comparison of nanoscaled and bulk NiO structural and environmental characteristics by XRD, XAFS, and XPS. Chem. Mater. 2012, 24 (23), 4483–4490. 10.1021/cm300739y.

