# Supplementary material for: Surfactant-Assisted Synthesis of Metallic-Ag/Nickel Oxide on Graphitic Carbon Nitride Composite: An Electrochemical Investigation of Synthetic Vanillin
Source: ACS Appl Mater Interfaces. 2025 Feb 6;17(7):11287–99. doi: 10.1021/acsami.4c19099 (PMC11843540; doi:10.1021/acsami.4c19099)
Supplement: Supplementary file 1 — am4c19099_si_001.pdf [file am4c19099_si_001.pdf]

Supporting Information

Surfactant-Assisted Synthesis of Metallic-Ag/Nickel Oxide on Graphitic Carbon Nitride Composite: An Electrochemical Investigation of Synthetic Vanillin

Muthukumar Govindaraj[a], Balasubramanian Sriram[b], Sea-Fue Wang[b], Magesh Kumar Muthukumaran[a], Sakthivel Kogularasu[c,d], Guo-Ping Chang-Chien[c,d,e], Arockia Selvi J[a,*]

[a] Department of Chemistry, SRM Institute of Science and Technology, Kattankulathur-603203, Tamil Nadu, India.

[b]Department of Materials and Mineral Resources Engineering, National Taipei University of Technology, Taipei 106, Taiwan.

[c]Super micro mass research and technology center, Cheng Shiu University, Kaohsiung 833301, Taiwan.

[d]Center for Environmental Toxin and Emerging-Contaminant Research, Cheng Shiu University, Kaohsiung 833301, Taiwan.

[e]Institute of Environmental Toxin and Emerging-Contaminant, Cheng Shiu University, Kaohsiung 833301, Taiwan.

***Corresponding author**

Email: arockiaj@srmist.edu.in (Arockia Selvi J)

No. of Pages: 7

No. of Figures: 1

No. of Table: 1

Table of content entry

Content entry		Page No.
Chemicals and reagents		Page No. S-2
Materials characterization		Page No. S-2
Figure S1	Cyclic stability of Ag/NiO/g-CN in the presence of vanillin	Page No. S-4
Table S1	Electro-analytical performance of Ag/NiO/g-CN/SPCE towards vanillin detection.	Page No. S-5
References		Page No. S-6

Chemicals and reagents

Silver nitrate ($AgNO_3$), nickel (II) nitrate hexahydrate ($Ni(NO_3)_2 \cdot 6H_2O$), hexamethylenetetramine (HMT- $C_6H_{12}N_4$), melamine ($C_3H_6N_6$) and urea ($CH_4 N_2 O$) are analytical grade and are received from Sigma-Aldrich & Merck, Alfa Aesar and Showa Chemical Industry Co., Ltd. Screen printed carbon electrode (SPCE) from Zensors.Pvt.Ltd, Taiwan. Ultrapure fresh water is obtained from a millipore water purification system (Milli-Q, specific resistivity > 18 MΩcm, S.A.; Molsheim, France) and is used in all the experiments. Sodium phosphate dibasic and sodium dihydrogen phosphate (Na_2HPO_4 and NaH_2PO_4) are utilized to prepare 0.1 M (pH 7) PB (phosphate buffer). All the electrochemical experiments are carried out using 0.1 M PB (pH 7) as the supporting electrolyte.

Materials characterization

High-resolution scanning electron microscopy (HR-SEM, Thermoscientific Apreo S) and transmission electron microscopy (TEM, JEM-2100 Plus, JEOL) were utilized to predict the morphological, surface properties, and chemical content, and the selected area electron diffraction (SAED) pattern of produced prepared materials. Energy dispersive spectroscopy

(EDS-JEM-2100 Plus, JEOL, and Thermoscientific Apreo S) was utilized for elemental and chemical composition. The crystalline nature of the synthesized compound was examined by using X-ray diffraction spectroscopy (XRD system-X'pert powder, with Cu-Kα radiation (λ = 0.154 nm) Malvern Panalytical, United Kingdom) at 45 kV (tension) and 40 mA (current) with 0.02° per step scan and 1° per min speed. The functional groups of the synthesized materials were analyzed by Fourier Transform Infrared Spectroscopy (FT-IR) technique, (FT-IR spectrophotometer, Shimadzu, IR Tracer 100). X-ray Photoelectron Spectroscopy (XPS) PHI VersaProbe III Scanning XPS Microprobe (Physical Electronics, USA) confirmed the chemical state, bonding nature, and binding energy. The electrochemical properties are explored using electrochemical impedance spectroscopy (EIS) through Autolab (PGSTAT101). The cyclic voltammetry (CV), and differential pulse voltammetry (DPV) studies were carried out using CHI electrochemical workstation (CHI 1211c) in a conventional three electrode cell. All the measurements were performed at room temperature using a three-electrode setup.

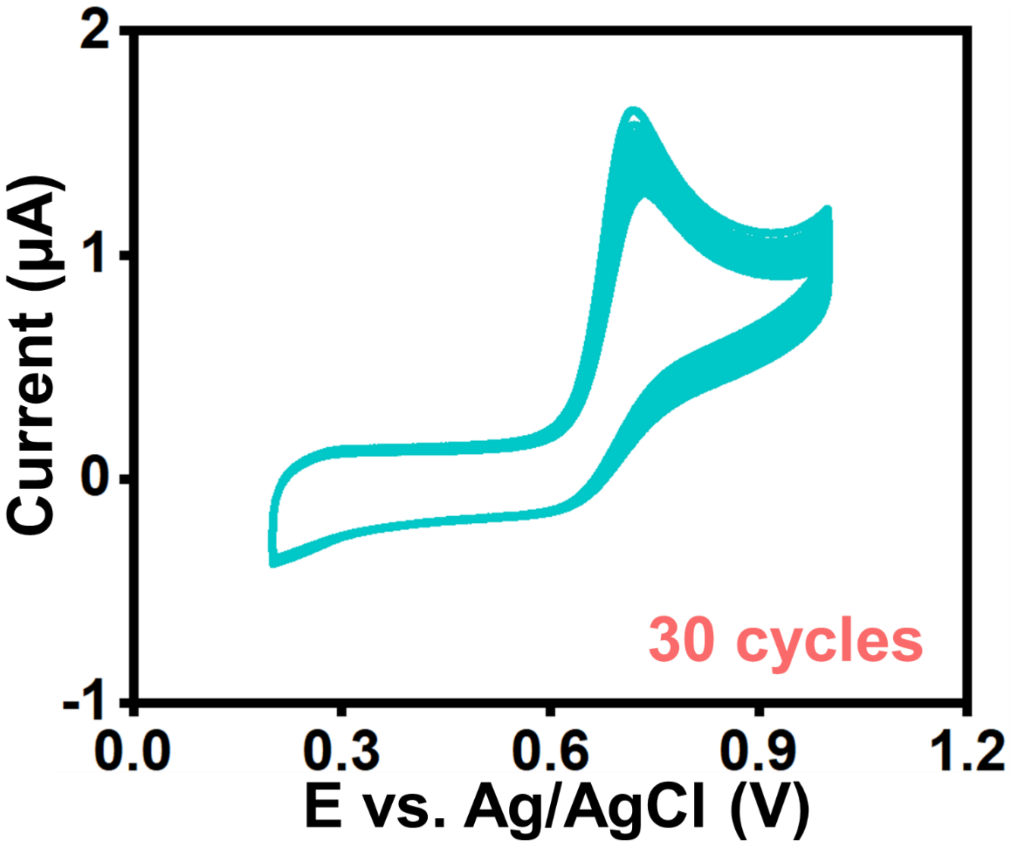

Figure S1. Cyclic stability of Ag/NiO/g-CN in the presence of vanillin.

Table S1. Electro-analytical performance of Ag/NiO/g-CN/SPCE towards vanillin detection.

Materials	Linear Range	LOD	Method	Ref.
AuPd-Gr/GCE	0.1–7.0; 10.0–40.0 μM	20.0 nM	DPV	S1
Gr–PVP/ABPE	0.02–2.0; 2.0–40.0; 40.0–100.0 μM	10.0 nM	Derivative voltammetry	S2
$g\text{-}C_3N_4$	0.02-10.0; 15.0–200.0 μM	4.0 nM	DPV	S3
$MoS_2/PANI/g\text{-}C_3N_4$	4.6–103.0 μM	43 nM	DPV	S4
Ni NPs-N-C	0.04–1.0; 1.0–10.0; 10.0–100.0 μM	10 nM	Derivative voltammetry	S5
FePc MOF	0.22–29.14 μM	50 nM	DPV	S6
Co_3O_4	0.02–209 μM	3 nM	DPV	S7
CMP-rGO	0.08– 33 μM	14 nM	DPV	S8
SnS/CNF	0.00125–506.75 μM	3.2 nM	i–t	S9
**Ag/NiO/g-CN**	**0.004–366.8 μM**	**0.9 nM**	**DPV**	**This work**

References

S1. Shang, L., Zhao, F. and Zeng, B., 2014. Sensitive voltammetric determination of vanillin with an AuPd nanoparticles− graphene composite modified electrode. *Food chemistry*, *151*, pp.53-57.

S2. Deng, P., Xu, Z., Zeng, R. and Ding, C., 2015. Electrochemical behavior and voltammetric determination of vanillin based on an acetylene black paste electrode modified with graphene–polyvinylpyrrolidone composite film. *Food chemistry*, *180*, pp.156-163.

S3. Fu, L., Xie, K., Wu, D., Wang, A., Zhang, H. and Ji, Z., 2020. Electrochemical determination of vanillin in food samples by using pyrolyzed graphitic carbon nitride. *Materials Chemistry and Physics*, *242*, p.122462.

S4. Murugan, E. and Dhamodharan, A., 2021. Separate and simultaneous determination of vanillin, theophylline and caffeine using molybdenum disulfide embedded polyaniline/graphitic carbon nitrite nanocomposite modified glassy carbon electrode. *Diamond and Related Materials*, *120*, p.108684.

S5. Nie, X., Deng, P., Zhang, R., Tang, Z., Wang, H. and Tang, Y., 2020. Nickel nanoparticles supported on nitrogen-doped carbon for vanillin detection. *ACS Applied Nano Materials*, *3*(12), pp.11791-11800.

S6. Nie, X., Deng, P., Zhang, R., Tang, Z., Wang, H. and Tang, Y., 2020. Nickel nanoparticles supported on nitrogen-doped carbon for vanillin detection. *ACS Applied Nano Materials*, *3*(12), pp.11791-11800.

S7. Lee, Y.Y., Sriram, B., Wang, S.F., Stanley, M.M., Lin, W.C., Kogularasu, S., Chang-Chien, G.P. and George, M., 2024. Eco-innovative electrochemical sensing for precise detection of

vanillin and sulfadiazine additives in confectioneries. *Applied Surface Science Advances*, *20*, p.100584.

S8. Li, X., Fang, Y., Li, H. and Feng, S., 2024. Heterojunction of branched benzopyrazine-based polymers coating on graphene for electrochemical sensing of vanillin. *Talanta*, *277*, p.126420.

S9. Gokulkumar, K., Huang, S.J., Lee, Y.Y., Kogularasu, S. and Chang-Chien, G.P., 2024. Nanoparticles of SnS on Carbon Nanofibers for Electrochemical Detection of Vanillin. *ACS Applied Nano Materials*, 7, 11, 13183–13193.

S10.
